# Autophagy suppression via SRC induction represents a therapeutic vulnerability for *BAP1*-mutant cancers

**DOI:** 10.1080/15548627.2025.2535265

**Published:** 2025-08-03

**Authors:** Silvia Vega-Rubin-de-Celis, Amanda Kristani, Matthias Kudla, Svenja Mergener, Andrés Corrochano-Ruiz, Safa Larafa, Jetsy Montero-Vergara, Laura-Marie Ahle, Rainer Will, Mael Lever, Viktor Grünwald, Boris Hadaschik, Verena Jendrossek, Nikolaos E. Bechrakis, Samuel Peña-Llopis

**Affiliations:** a Institute for Cell Biology (Cancer Research), University Hospital Essen, Essen, Germany; b Translational Genomics, German Cancer Consortium (DKTK) at University Hospital Essen, Essen, Germany; c Department of Ophthalmology, University Hospital Essen, Essen, Germany; d German Cancer Consortium (DKTK) and German Cancer Research Center (DKFZ), Heidelberg, Germany; e Core Facility Cellular Tools, German Cancer Research Center (DKFZ), Heidelberg, Germany; f Department of Urology, University of Duisburg-Essen, and German Cancer Consortium (DKTK), University Hospital Essen, Essen, Germany; g Department of Medical Oncology, University Hospital Essen, Essen, Germany

**Keywords:** BRCA1-associated protein 1, personalized medicine, patient-derived organoids, PDOs, tumoroids, cholangiocarcinoma

## Abstract

BAP1 is a tumor suppressor and epigenetic modifier that is frequently mutated in cancer, leading to increased aggressiveness and metastasis, as well as poor patient survival. Unfortunately, there are currently no specific therapies for metastatic tumors harboring *BAP1* mutations. In this study, we have identified a new targetable BAP1-associated autophagic vulnerability. We demonstrate that BAP1 transcriptionally regulates the proto-oncogene SRC, a non-receptor tyrosine kinase. SRC then binds to, phosphorylates, and inactivates BECN1 (Beclin 1), an essential autophagy protein. This inhibits autophagy in cells derived from various cancer types with *BAP1* mutations. Treatment of these cells with SRC inhibitors (such as dasatinib, bosutinib and saracatinib) and autophagy-inducing drugs (such as Tat-BECN1, SW076956 and SW063058) demonstrated a synergistic interaction between these compounds both *in vitro* and *in ovo* using a chick Chorioallantoic Membrane (CAM) assay. Furthermore, *ex vivo* studies employing patient-derived tumor organoids (PDTOs) of uveal melanoma (UM) and clear-cell renal cell carcinoma (ccRCC) as preclinical models have substantiated the synergism of these drugs, preferentially in the context of BAP1 loss. Our findings elucidate a novel BAP1-SRC-BECN1-autophagy regulatory axis that can be exploited therapeutically in precision oncology through the combination of SRC inhibitors and autophagy inducers, contingent upon patient stratification for BAP1 loss.

**Significance**: Deadly cancers with *BAP1* mutations suppress autophagy by phosphorylating the autophagy regulator BECN1 via the proto-oncogene SRC. Treatment with SRC inhibitors and autophagy inducers exhibited synergism *in* *vitro*, *in ovo* and in patient-derived tumor organoids with BAP1 loss, paving the way for treating BAP1-deficient cancers with autophagy inducers and kinase inhibitors.

## Introduction

BRCA1-associated protein 1 (*BAP1*) is a ubiquitin carboxy-terminal hydrolase that works as part of the Polycomb Repressive-Deubiquitinase (PR-DUB) complex [1]. BAP1 de-ubiquitinates histone H2A, thereby remodeling chromatin and maintaining the functional epigenetic landscape [1]. The enzymatic activity of BAP1 in the PR-DUB complex directly opposes the histone ubiquitylation of Polycomb Repressive Complex 1 (PRC1), regulating gene expression and various cellular processes, including DNA double-strand break repair [2], DNA synthesis [3], cell cycle progression [4] and metabolism [5]. *BAP1* has been found to be mutated in 80% of metastatic uveal melanoma (UM) [6], 63% of malignant pleural mesothelioma [7,8], 25% of intrahepatic cholangiocarcinoma [9], 15% of clear-cell renal cell carcinoma (ccRCC) [10], and at lower frequencies in other cancers. *BAP1* mutations are always accompanied by partial or complete loss in chromosome 3, where the *BAP1* gene is located [11,12]. BAP1 loss is associated with higher tumor aggressiveness [10], metastasis [6] and poor patient survival [13–15]. Indeed, *BAP1* is the cancer gene most frequently associated with poor patient survival across many common tumor types [16].

We have previously shown that ccRCC tumors can be classified into those with mutually exclusive mutations in *BAP1* (and associated with high tumor grade, activation of mTORC1 and poor patient survival) and mutations in *PBRM1* (and associated with better prognosis) [10,11,13]. UM tumors can be classified into those with mutually exclusive mutations in *EIF1AX* or *SF3B1* (and associated with better prognosis) and tumors with mutations in *BAP1* [14]. In contrast to metastatic cutaneous melanoma, metastatic UM shows limited response to immune checkpoint blockade, in part due to the unique immune system in the eye and the different connection to the vascular system [17]. Despite clinical trials using conventional chemotherapy, immunomodulatory, or targeted therapies, no reliable treatment is currently available for metastatic UM. Since 2022, the T-cell engager tebentafusp is the first and only approved therapy for the treatment of unresectable or metastatic UM [18].

A study of uveal melanoma (UM) samples from patients at various stages of the disease identified a possible correlation between macroautophagy (hereafter referred to as autophagy, self-eating) and hypoxic tumor regions, suggesting that autophagy may play a role in UM progression [19]. Autophagy is a lysosomal degradation pathway involved in a variety of physiological and pathological conditions, including protection against diseases, such as cancer [20]. The role of autophagy in cancer is rather complex and context-dependent [21], and although mutations in autophagy (*Atg*) genes are detected in some cancers [22,23], there is no evidence suggesting that these are drivers of tumorigenesis. Autophagy is deregulated in multiple cancers and it is plausible to hypothesize that restoring cellular homeostasis to its normal levels may be more critical in tumor cells than in normal cells. Numerous clinical trials using drugs that inhibit autophagy (mostly chloroquine and hydroxychloroquine) in combination with chemotherapy or radiation therapy have been reported in multiple types of cancer [24–26], but unfortunately, the results have been inconclusive so far. These disappointing outcomes are not entirely unexpected due to several factors, including the complexity of the disease, the non-specificity of the drugs used (chloroquine and hydroxychloroquine inhibit all lysosomal functions, not only autophagy), the activation of metastatic dormant cells upon autophagy inhibition, and the fact that autophagy can play a cytoprotective or a cytotoxic role in different contexts [27]. Induction of autophagy, despite its pro-survival role in some cancers, may also be considered as a therapeutic approach due to its cytotoxic and tumor suppressor effects, particularly in cancers that accumulate abnormal proteins or damaged organelles [28]. Therefore, it is essential to identify which tumors may be more susceptible to autophagy modulating drugs by analyzing various factors, including genetic background, oncogenic mutations, metabolic profile, microenvironment and tissue of origin.

Here, we identified an autophagic vulnerability in cancers with *BAP1* mutations involving the oncogenic tyrosine kinase SRC and the autophagy protein BECN1 that can be therapeutically targeted, opening the possibility of novel treatments specific for tumors with BAP1 loss.

## Results

### BAP1 regulates SRC levels transcriptionally

**Figure 1.**
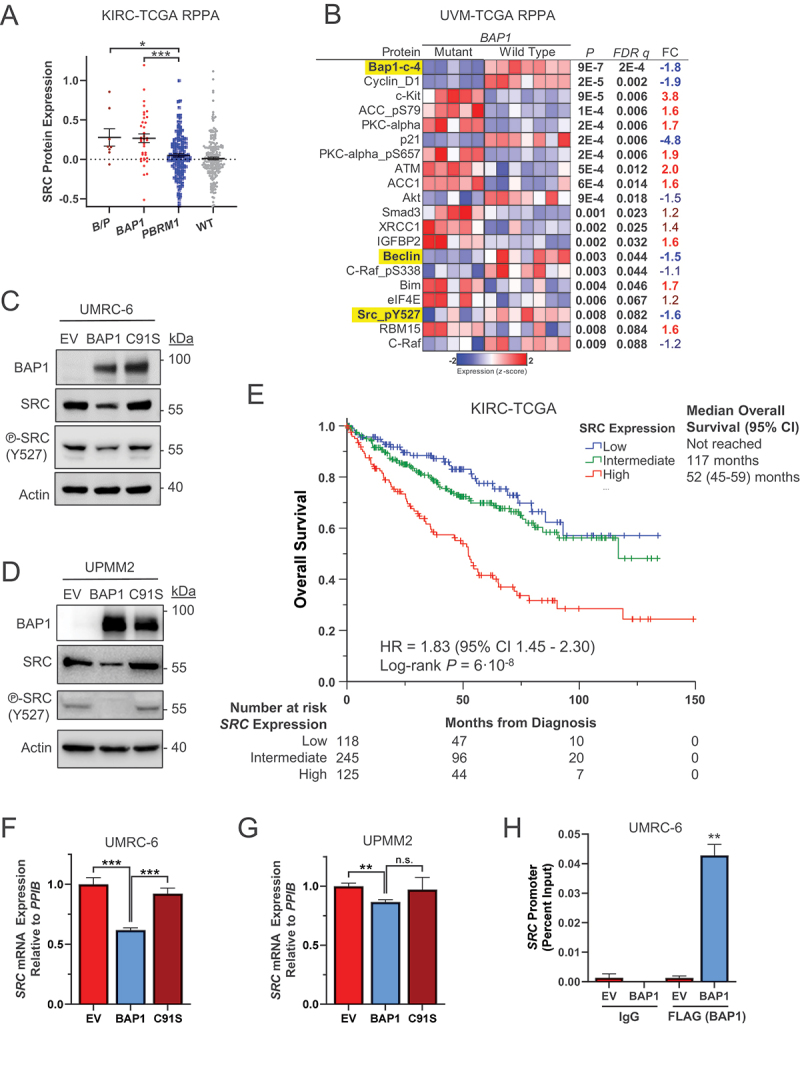
BAP1 regulates SRC transcriptionally. (A) reverse-phase protein array (RPPA) data analysis of SRC in KIRC-TCGA (clear cell renal cell carcinoma, ccRCC) stratified by *BAP1* and/or *PBRM1* mutation status. (B) RPPA data analysis in UVM-TCGA (uveal melanoma, UM) stratified by *BAP1* mutation status. (C,D) Representative western blotting analysis of BAP1-deficient ccRCC UMRC-6 cell lines (C) and UM UPMM2 cell lines (D) reconstituted with an empty vector (EV), wild-type *BAP1*, or p.C91S catalytically inactive *BAP1* mutant. (E) Kaplan-Meier curves of overall survival in KIRC-TCGA depending on *SRC* expression levels, stratified by quartile. Intermediate *SRC* expression is the combination of the second and third quartiles. (F,G) qRT-PCR analysis of *SRC* mRNA expression in these reconstituted BAP1-deficient ccRCC (F) and UM (G) cell lines. (H) chromatin immunoprecipitation (ChIP) to assess BAP1 occupancy on the *SRC* promoter. Error bars represent the average ± SE (*n* = 3–4). HR, hazard ratio; CI, confidence interval; *n.s*., non-significant; *, *p* < 0.05; **, *p* < 0.01; ***, *p* < 0.001 for the indicated comparisons by *t*-tests.

To gain insight into the mechanism of increased aggressiveness of *BAP1*-mutant tumors, we performed bioinformatics analyses on The Cancer Genome Atlas (TCGA). Reverse-phase protein array (RPPA) on KIRC-TCGA curated data showed that one of the most upregulated proteins in *BAP1*-mutant tumors from ccRCC patients is the non-receptor tyrosine kinase SRC, and it also showed a significant decrease in the phosphorylation levels of the inhibitory SRC site Y527 (Figure 1A and S1A). A similar analysis of RPPA data from uveal melanoma (UM) tumors from TCGA (UVM-TCGA) also showed that tumors with *BAP1* mutations have a decrease in SRC phosphorylation at Y527 (Figure 1B). We confirmed these data by western blotting in a panel of BAP1-deficient cell lines from different entities reconstituted with an empty vector control or a vector encoding an HA-tagged wild-type *BAP1* (or a catalytically inactive p.C91S mutant) [29], including the ccRCC cell line UMRC-6 (Figure 1C and S1B), the UM cell line UPMM2 (Figure 1D and S1C), the cholangiocarcinoma cell line TFK-1 (Figure S1D,E), and the breast cancer cell line HCC-1187 (Figure S1F,G). In all cell lines tested, a significant decrease in SRC levels was detected when cells expressed the wild-type form of BAP1, but not when cells expressed the catalytically inactive form of BAP1 (p.C91S), indicating that an active form of the epigenetic modulator BAP1 is required for regulation of SRC levels and, suggesting transcriptional regulation. Furthermore, decreased SRC Y527 phosphorylation was also observed in cell lines reconstituted with the wild-type form of BAP1 but not in those expressing the catalytically inactive BAP1 p.C91S (Figure 1C,D and S1D), similar to the pattern observed in RPPA data from both UM (Figure 1B) and ccRCC tumors (Figure S1A).

In light of our prior findings demonstrating that ccRCC patients with *BAP1* mutations exhibit adverse outcomes with respect to both prognosis [10] and overall survival [13], we sought to ascertain whether BAP1 levels exhibit a correlation with SRC levels. Indeed, our investigation revealed a negative correlation between BAP1 protein expression, as determined by RPPA, and both mRNA and protein SRC levels (Figure S1H,I). This finding holds clinical significance, as ccRCC patients with high SRC expression exhibited a median overall survival of 52 months (95% confidence interval [CI] 45–59 months), which was significantly shorter than the 117-month survival observed in patients with intermediate SRC levels (*p* = 6·10^−8^) (Figure 1E). Similar results were also found in UM (Figure S1L).

To ascertain whether SRC protein levels are transcriptionally regulated by BAP1, *SRC* mRNA levels were determined by qRT-PCR in BAP1-deficient ccRCC cells (UMRC-6) and UM cells (UPMM2) (Figure 1F,G). Our results confirmed that *SRC* levels are regulated transcriptionally by BAP1, since reconstitution with wild type but not mutant *BAP1* decreases *SRC* expression. Furthermore, bioinformatics analysis of chromatin immunoprecipitation-sequencing (ChIP-Seq) data in bone marrow-derived macrophages in mice [30] indicates that BAP1, along with Hcf-1 and Ogt (members of the Bap1 multiprotein complex to regulate gene expression [1,31]), bind to the *Src* promoter in mice (Figure S1M) and might act as a negative regulator of *Src* expression, in agreement with our data. To further validate these findings, we designed primers for the human *SRC* promoter that target the area of binding of BAP1 to mouse *Src* promoter in ChIP-Seq. We then conducted ChIP-qPCR assays in UMRC-6 cells reconstituted with FLAG-BAP1 or empty vector. The obtained data indicate that BAP1 binds to the *SRC* promoter when BAP1 is expressed in cells (Figure 1H), demonstrating a direct regulation of *SRC* mRNA levels by BAP1.

To exclude the possibility of an additional post-translational regulatory level, the stability of the SRC protein in UMRC-6 cells reconstituted with wild-type BAP1 (or an empty vector control) was determined by blocking translation with cycloheximide over time. The results indicate that the half-life of SRC is similar in both cell lines (Figure S1J,K). Taken together, these data indicate that BAP1 regulates *SRC* transcriptionally.

### BAP1-loss-mediated SRC activation promotes autophagy suppression

**Figure 2.**
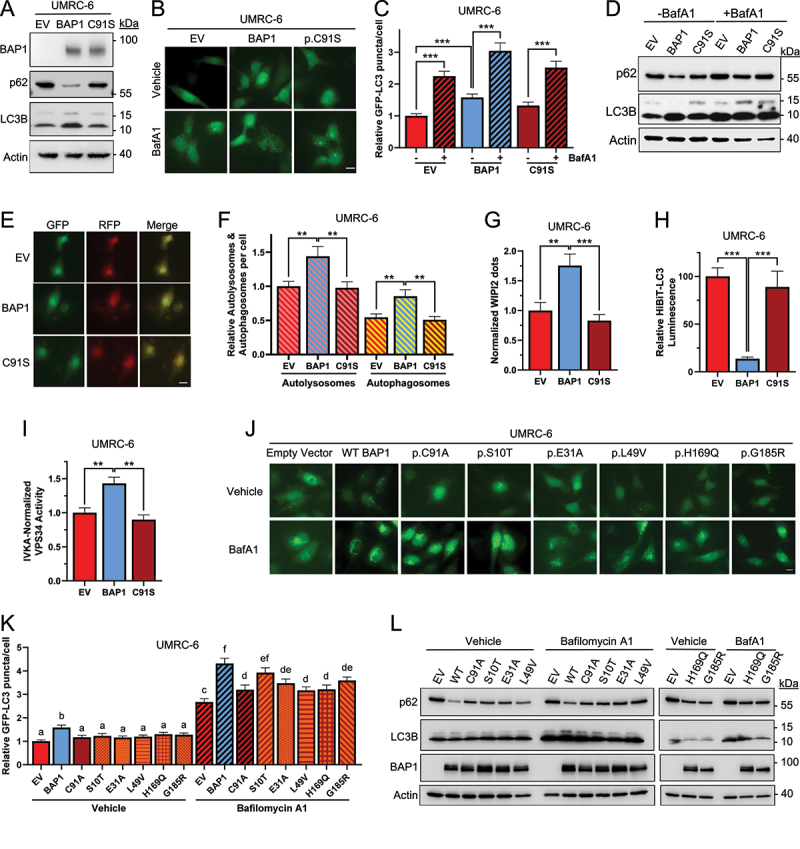
BAP1 loss suppresses autophagy. The autophagic activity of UMRC-6 cells reconstituted with an empty vector (EV) or *BAP1* (wild-type or p.C91S mutant) was analyzed using the following methods: (A) western blotting, (B,C) quantification of GFP-LC3 puncta (*n* = 50), (D) western blotting of samples treated with Bafilomycin A1 (BafA1; 100 nM, 3 h), (E,F) quantification of autolysosomes *vs*. autophagosomes, (G) the number of WIPI2 dots per cell, (H) quantification of HiBiT-LC3 luminescence, and (I) the kinase activity of BECN1-bound VPS34. (J-L) Autophagy in UMRC-6 cells reconstituted with an empty vector (EV), wild-type *BAP1*, or multiple *BAP1* mutants observed in tumors were analyzed by microscopy of GFP-LC3 puncta formation (J,K) and western blotting (L) in cells treated with Bafilomycin A1 (100 nM, 3 h) or the DMSO control (vehicle). Error bars represent the average ± SE of three independent experiments. *n.s*., non-significant; **, *p* < 0.01; ***, *p* < 0.001 for the indicated comparisons by *t*-tests. Non-overlapping letters (e.g., “a” *vs*. “b”) represent significant differences (*p* < 0.05) using ANOVA and Student-Newman-Keuls test. Scale bar: 20 μm.

**Figure 3.**
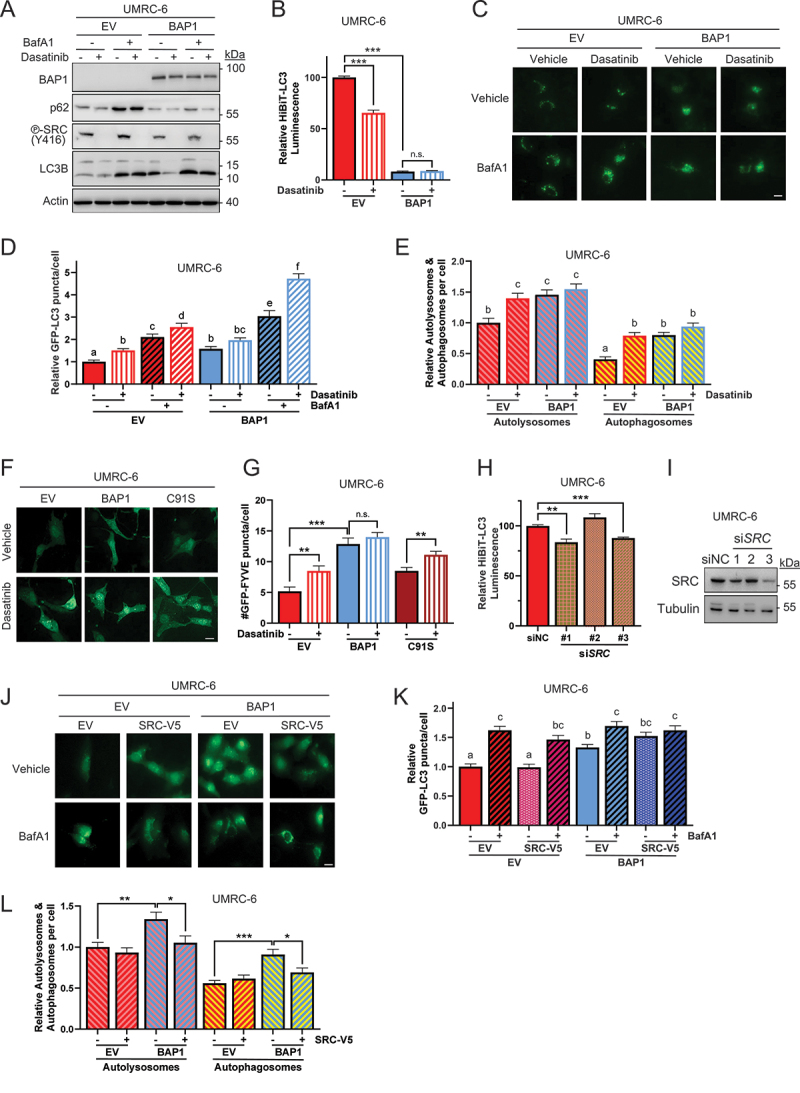
BAP1 loss leads to SRC-mediated suppression of autophagy. The effects of SRC inhibition on autophagy were assessed by treatment with dasatinib, and samples were analyzed by (A) western blotting, (B) HiBiT-LC3 luminescence, (C) GFP-LC3 puncta and (D) their quantification (*n* = 50), (E) number of autophagosomes and autolysosomes (*n* = 50), (F,G) number of GFP-FYVE puncta (*n* = 50) treated with 1 µM dasatinib for 24 h or DMSO control, as well as by *SRC* silencing (H,I) or overexpression of a V5-tagged *SRC* construct (J-L). Bafilomycin A1 was added at 100 nM for 3 h and dasatinib at 1 µM for 24 h. Error bars represent the average ± SE of three independent experiments. *n.s*., non-significant; *, *p* < 0.05; **, *p* < 0.01; ***, *p* < 0.001 for the indicated comparisons by *t*-tests. Non-overlapping letters represent significant differences (*p* < 0.05) by ANOVA and Student-Newman-Keuls test. Scale bar: 20 μm.

**Figure 4.**
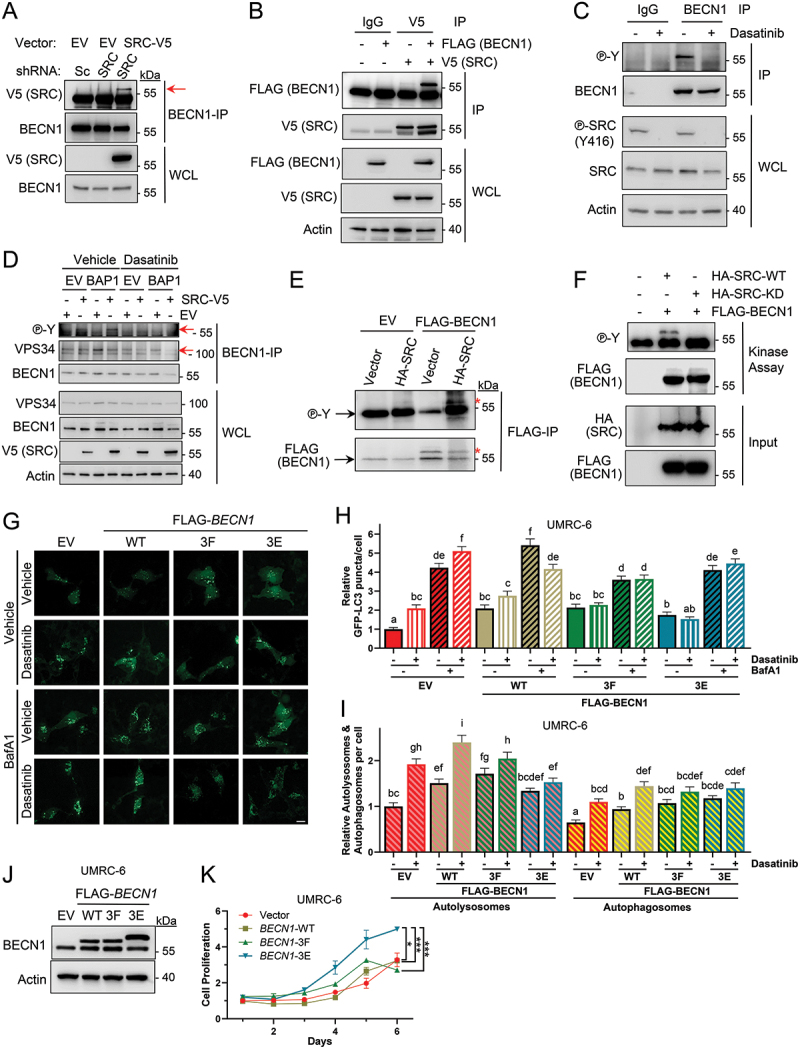
SRC binds to and phosphorylates BECN1 to regulate autophagy and proliferation. (A) BECN1 immunoprecipitation (IP) in UMRC-6 cells that overexpress *SRC*-V5 or an empty vector control (EV), containing plasmids for either constitutive *SRC* knockdown (sh*SRC*) or a scrambled control (shSc). The arrow indicates the *SRC*-V5 band, and the bands below correspond to the heavy chain of IgG. (B) *SRC*-V5 was immunoprecipitated from UMRC-6 cells that overexpress *SRC*-V5 and were transfected with FLAG-BECN1 (or the corresponding empty vector control). (C) BECN1 (or IgG control) IP of UMRC-6 cells, followed by western blot analysis of phosphorylated tyrosine residues. (D) BECN1 IP of UMRC-6 cells overexpressing *SRC*-V5 (or empty vector control, EV) along with BAP1 (or EV) treated with 1 µM dasatinib for 24 h or DMSO control (vehicle). Red arrows indicate the bands for phosphorylated BECN1 (top) and VPS34 (bottom). (E,F) *in vitro* kinase assays of FLAG-BECN1 (or vector control) using *in vitro* transcribed/translated HA-SRC (E) or a kinase-dead version (F). The arrows in (E) indicate IgG heavy chains and the red asterisks (*) indicate the phosphorylated (top) and total (bottom) BECN1. (G-K) UMRC-6 cells stably expressing wild-type (WT) FLAG-BECN1, tyrosine phosphorylation mutants (non-phosphorylatable [BECN1 Y229/233/352F, BECN1-3F] or phospho-mimetic [BECN1 Y229/233/352E, BECN1-3E]) or an empty vector control (EV) were analyzed for autophagic flux by the number of GFP-LC3 puncta (G,H), the number of autolysosomes and autophagosomes (I), western blotting (J) or cell proliferation by Hoechst staining of the nuclei (K). Baf A1, 100 nM Bafilomycin A1 for 3 h; IP, immunoprecipitation; WCL, whole cell lysate. Error bars represent the average ± SE (*n* = 50). *n.s*., non-significant; *, *p* < 0.05; ***, *p* < 0.001 by *t*-tests. Non-overlapping letters represent significant differences (*p* < 0.05) by ANOVA and Student-Newman-Keuls test. Scale bar: 20 μm.

**Figure 5.**
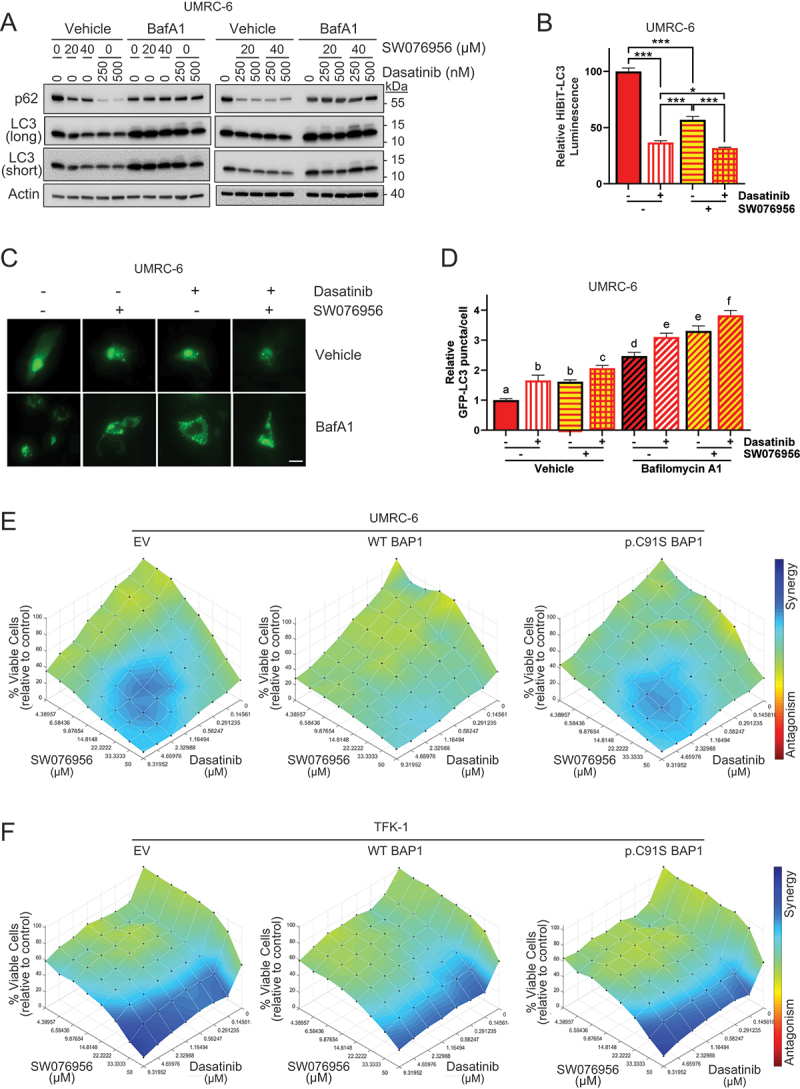
Dasatinib and SW076956 synergistically induce autophagy and decrease cell viability *in vitro*. (A-D) the effects of dasatinib (at nM concentration) and SW076956 (at µM concentration) treatments on autophagy in UMRC-6 cells were assessed by western blotting alone (left panel) or in combination (right panel) (A), HiBiT-LC3 luminescence (B), and the number of GFP-LC3 puncta (*n* = 50) (C,D). Cells were treated with 1 µM dasatinib and/or 40 µM SW076956 for 24 h. Baf A1, 100 nM Bafilomycin A1 for 3 h. Error bars represent the average ± SE of three independent experiments. *n.s*., non-significant; *, *p* < 0.05; ***, *p* < 0.001 by *t*-tests. Non-overlapping letters indicate significant differences (*p* < 0.05) by ANOVA and Student-newman-keuls test. (E,F) the combination effects of dasatinib and SW076956 treatments on cell viability of UMRC-6 (E) or TFK-1 (F) cells reconstituted with empty vector (EV), wild-type *BAP1*, or a p.C91S *BAP1* mutant were quantified 72 h after treatment of serial dilutions of single and combined compounds for three independent experiments. The synergy/antagonism effects of dasatinib and SW076956 were determined using a Loewe synergy model with Combenefit software from three independent experiments.

Oncogenic tyrosine kinases are involved in the regulation of autophagy in several cancers through BECN1 (BCL2 interacting myosin like coiled protein) phosphorylation [32], including EGFR (epidermal growth factor receptor) in non-small cell lung cancer [33], HER2/ERBB2 (human epidermal growth factor receptor 2) in breast cancer [34], BCR-ABL (breakpoint cluster region-ABL proto-oncogene 1 non-receptor tyrosine kinase) in chronic myeloid leukemia [35], and c-KIT in Merkel cell carcinoma [36]. Tyrosine phosphorylation of BECN1 at residues Y229, Y233 and Y352 shifts the equilibrium from the active, complexed BECN1 within the class III phosphatidylinositol (PI) 3-kinase complex 1 (PI3KC3-C1) to an inactive, homodimerized form in which autophagic flux is inhibited [32]. Since SRC is also a tyrosine kinase (albeit a non-receptor tyrosine kinase), we hypothesized that it might be similarly involved in autophagy regulation through BECN1 phosphorylation, and that increased SRC levels due to BAP1 loss might also have an inhibitory effect on autophagy. Therefore, we first evaluated the basal autophagy activity in UMRC-6 cells reconstituted with an empty vector, wild-type *BAP1* or the p.C91S mutant. Indeed, cells expressing the wild-type form of *BAP1* had an elevated basal autophagy activity compared to the other cells, as determined by 1) lower levels of the autophagy substrate p62 and increased levels of LC3B-II by western blotting (Figure 2A,D); 2) increased levels of the autophagosome-localized GFP-LC3 reporter detected by the GFP-LC3 puncta formation assay; the number of puncta is further increased with the addition of the lysosomal inhibitor Bafilomycin A1 (BafA1), indicating activation of autophagic flux rather than a block in autophagosome maturation (Figure 2B,C); 3) increased numbers of autolysosomes as assessed by the red signal of the mRFP-GFP-LC3B dual reporter; the GFP signal is quenched upon lysosomal fusion to the autophagosome and therefore an increase in autophagic flux is reflected only as red puncta (Figure 2E,F); 4) increased number of WIPI2 dots (Figure 2G); 5) decreased luminescence of the HiBiT-LC3 reporter, which ultimately measures total LC3 levels [37] (Figure 2H); and 6) increased activity of BECN1-bound VPS34 activity determined by *in vitro* kinase assay (Figure 2I). Taken together, these data demonstrate that UMRC-6 cells with loss of BAP1 have lower autophagy activity, which is activated upon reconstitution with wild-type *BAP1*, but not the catalytically inactive p.C91S *BAP1* mutant. Interestingly, the regulation of the autophagy substrate p62/*SQSTM1* seems to be rather complex in this context, since there is a transcriptional regulation of its mRNA levels (determined by qRT-PCR) by BAP1 (Figure S2I-K). Basal p62 expression levels are decreased upon BAP1 reconstitution, and these correlate with protein levels. However, when cells are treated with Bafilomycin A1, a similar accumulation of p62 is observed in all cells, whereas no significant change occurs at the mRNA level. In addition, UMRC-6 cells reconstituted with *BAP1* mutants found in cancer patients [5] indicate that only the wild-type *BAP1* is able to induce autophagy (Figure 2J–L and S2L,M), therefore, suggesting that all clinically relevant *BAP1* mutations analyzed behave as loss-of-function in their ability to modulate autophagy. Similar results were found in BAP1-deficient cholangiocarcinoma TFK-1 cells (Figure S2A-C), UM UPMM2 cells (Figure S2D,E), breast cancer HCC-1187 cells (Figure S2F,G), and ccRCC 786–0 cells with wild-type *BAP1* knocked out using two different CRISPR-Cas9 guided-RNAs (Figure S2H).

To ascertain whether the observed changes in autophagic flux were indeed correlated with SRC activity, we performed experiments modulating SRC activity by either inhibiting (by siRNA knockdown or by treatment with dasatinib, a SRC inhibitor) or overexpressing *SRC* (using a V5-tagged *SRC* construct) in BAP1-deficient ccRCC UMRC-6 cells. SRC inhibition by dasatinib induced autophagic flux as determined by decreased p62 levels by western blotting (Figure 3A); decreased luminescence of the HiBiT-LC3 reporter (Figure 3B); increased GFP-LC3 puncta (Figure 3C,D); increased numbers of autolysosomes as determined by the red signal of the mRFP-GFP-LC3B dual reporter (Figure 2E); and increased activity of the catalytic subunit of the Class III PI3KC1, VPS34, as assessed by the number of GFP-FYVE puncta (Figure 3F,G) in UMRC-6 cells treated with dasatinib. Further HiBiT-LC3 and GFP-LC3 puncta analysis with other SRC inhibitors (bosutinib and saracatinib) confirmed such induction of autophagic flux upon SRC inhibition (Figure S3B,C). Furthermore, SRC depletion by siRNA also induced autophagic flux in UMRC-6 cells as assessed by the HiBiT-LC3 reporter (Figure 3H,I). Therefore, genetic or pharmacological inhibition of SRC induced autophagy in BAP1-deficient cells at least in part through modulation of the VPS34 activity.

Furthermore, overexpression of a V5-tagged *SRC* significantly increased the number of GFP-LC3 puncta per cell in UMRC-6-BAP1 cells, although these numbers did not increase upon addition of Bafilomycin A1, suggesting an inhibition of the autophagosome-lysosomal fusion (Figure 3J,K). In contrast, the empty vector control on the BAP1 reconstituted cells further increased the GFP-LC3 levels upon addition of Bafilomycin A1. Furthermore, the increased number of autolysosomes observed in cells reconstituted with BAP1 decreased when V5-SRC was overexpressed, as assessed by the red signal of the mRFP-GFP-LC3B dual reporter, suggesting a SRC-dependent modulation of autophagic flux (Figure 3L).

Taken together, these data strongly suggest that SRC is both necessary and sufficient to modulate autophagy activity in BAP1-deficient cells.

### SRC binds to and phosphorylates BECN1

Autophagy is regulated by multiple signals through the PI3KC3-C1 complex, including BECN1 phosphorylation by several kinases [38]. To test whether SRC might regulate autophagy through binding and phosphorylation of BECN1, we generated UMRC-6 cells containing a shRNA targeting endogenous *SRC* (or a non-targeting shRNA control) and a plasmid overexpressing a V5-tagged *SRC* (or empty vector, EV, control). We found that *SRC*-V5 co-immunoprecipitated with endogenous BECN1 (Figure 4A). In addition, FLAG-BECN1 was also immunoprecipitated with *SRC*-V5 in cells overexpressing *SRC*-V5 and FLAG-BECN1 (Figure 4B).

Furthermore, analysis of BECN1 tyrosine phosphorylation also showed that endogenous BECN1 is phosphorylated at tyrosine residues in UMRC-6 cells (Figure 4C) and that this phosphorylation decreases upon treatment with dasatinib as determined by IP-WB. In addition, overexpression of *SRC*-V5 in both UMRC-6-EV and UMRC-6-BAP1 cells increased BECN1 tyrosine phosphorylation, and such phosphorylation was abolished by dasatinib treatment (Figure 4D). BAP1 reconstitution in UMRC-6 cells increased the amount of VPS34 immunoprecipitated with BECN1 (Figure 4D), consistent with data from the GFP-2xFYVE assays (Figure 3E,F), where more GFP dots are detected and VPS34/BECN1 binding decreases when *SRC*-V5 is overexpressed. To address the potential direct phosphorylation of BECN1 by SRC we performed *in vitro* kinase assays of FLAG-BECN1 purified from UMRC-6 stably expressing FLAG-BECN1 (or an empty vector control) with *in vitro* transcribed-translated HA-SRC (or a vector control). Western blot analysis of phosphorylated FLAG-BECN1 showed a band only on the samples containing FLAG-BECN1 and HA-SRC (Figure 4E). Furthermore, a kinase-dead mutant of HA-SRC (p.K298M) was unable to phosphorylate BECN1 (Figure 4F), indicating that the detected band was indeed specific for SRC phosphorylation. Taken together, these data strongly suggest that BECN1 co-immunoprecipitates with SRC and that BECN1-tyrosine phosphorylation in this context is regulated by SRC activity.

To determine whether the autophagic flux of UMRC-6 cells lacking BAP1 depends on the phosphorylation status of BECN1, we generated stable cell lines that overexpress FLAG-BECN1-WT or two BECN1 mutants with non-phosphorylatable substitutions (BECN1 Y229/233/352F, hereafter referred to as BECN1-3F) or phosphomimetic mutations (BECN1 Y229/233/352E, hereafter referred to as BECN1-3E) in the tyrosine residues involved in BECN1 dimerization and inactivation upon tyrosine phosphorylation [32,33]. Autophagic flux was assessed by measuring GFP-LC3 levels in each cell line (Figure 4G,H) and by counting autophagosomes/lysosomes using the mRFP-GFP-LC3B reporter (Figure 4I). Our results suggest that overexpression of BECN1-WT induces autophagic flux, which is further increased by dasatinib treatment. However, cells overexpressing the BECN1 mutants were unable to induce autophagy upon dasatinib treatment, indicating that the aforementioned BECN1 residues are required for the autophagic response to dasatinib.

### Proliferation of BAP1-deficient UMRC-6 cells is dependent on BECN1 tyrosine phosphorylation status

To investigate the impact of BECN1 phosphorylation on the proliferation of BAP1-deficient UMRC-6 cells, we employed stable cell lines that express either the unphosphorylatable (3F) or the phosphomimetic (3E) forms of BECN1. We found that cells overexpressing BECN1-3E, which mimics the status of constitutively phosphorylated BECN1, grow significantly faster than cells stably expressing wild-type or the 3F form of BECN1 (or an empty vector control) (Figure 4J,K). We confirmed these results using a colony formation assay, which revealed a higher number of colonies in cells overexpressing BECN1-3E (Figure S3J,K). These data suggest that the proliferation of the BAP1-deficient ccRCC cell line, UMRC-6, depends on the tyrosine phosphorylation status of BECN1. This finding suggests that BECN1-dependent processes, such as autophagy, play a crucial role in this context.

### SRC inhibition and autophagy induction are synergistic *in vitro*

**Figure 6.**
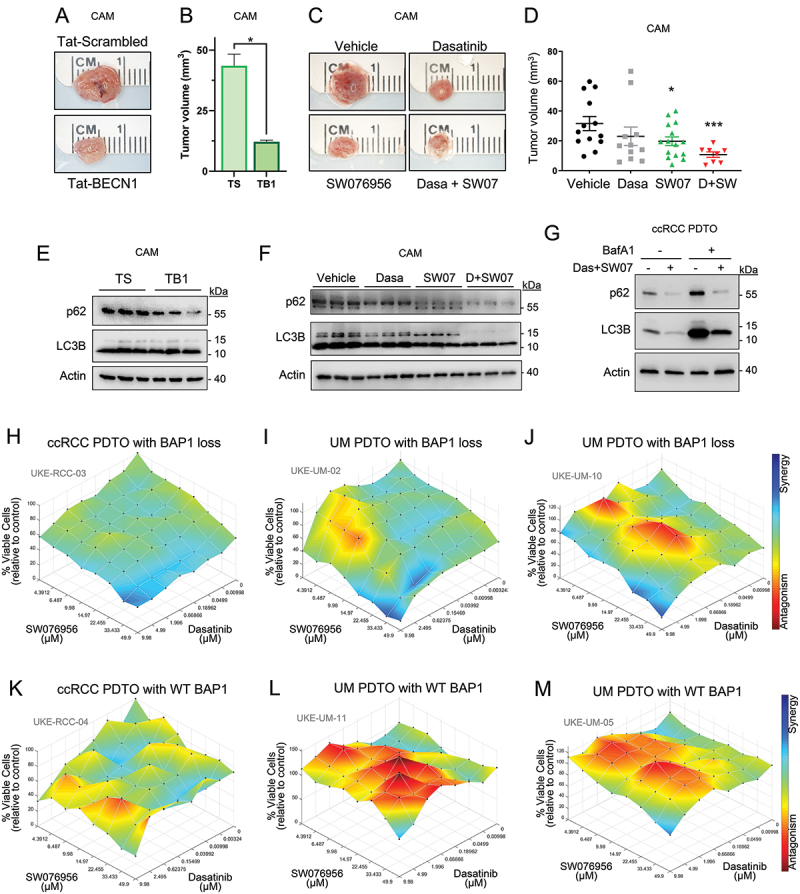
Dasatinib and SW076956 synergistically induce autophagy and reduce tumor growth *in ovo* and *ex vivo* in patient-derived tumor organoids. (A-D) Representative tumors of chorioallantoic membrane (CAM) assay (A) and quantification of tumor growth (B) of tumors treated with 10 µM Tat-BECN1 peptide (TB1) or 10 µM Tat-scrambled control (TS) (A,B) or with dasatinib (dasa, 1 µM), SW076956 (SW07, 40 µM) or a combination of both (C,D) for 7 days. (E-G) western blot analysis of several tumors treated with the TB1 or TS peptides (E), dasatinib, SW07 or a combination (F), and a representative patient-derived tumor organoid treated with the indicated compounds (G); BafA1: bafilomycin A1, 100 nM, 3 h; Das+SW07, combination of 1 µM dasatinib and 40 µM SW076956 for 24 h. Error bars represent the average ± SE. *, *p* < 0.05; ***, *p* < 0.001, *t*-test. (H-M) ombination effects of treatment with different concentrations of dasatinib and SW076956 on the viability of patient-derived tumor organoids (PDTOs) of ccRCC with BAP1 loss (H), ccRCC PDTOs with wild-type BAP1 (K), UM PDTOs with BAP1 loss (I,J) and UM PDTOs with wild-type BAP1 (L,M). The synergy/antagonism effects were determined using a Loewe synergy model with Combenefit software from two (I-M) or three (H) independent experiments.

Autophagy induction has been shown to be beneficial in breast cancers with amplifications in the receptor tyrosine kinase HER2/ERBB2. This has been demonstrated by preventing tumor formation in a genetically modified mouse model with higher basal autophagy levels and by decreasing tumor growth in HER2+ breast cancer xenografts treated with Tat-BECN1, an autophagy-inducing peptide [34]. Based on our observations of autophagy flux modulation in BAP1-deficient cells compared to reconstituted cells, we investigated whether treating BAP1-deficient UMRC-6 cells with Tat-BECN1 would induce autophagy *in vitro*. We confirmed that Tat-BECN1 induced autophagy in UMRC-6 cells using the HiBiT-LC3 luminescence assay and Western blot analysis of p62 levels (Figure S3F,G). To determine whether this treatment affects tumor growth, we used an *in ovo* approach in which tumor cells are implanted into the chorioallantoic membrane of fertilized chicken eggs. This approach replaces the use of mice for experimentation. The chorioallantoic membrane is highly vascularized, providing tumor cells with the necessary oxygen and nutrients to survive. Tumor samples treated with Tat-BECN1 were significantly smaller than those treated with the Tat-Scrambled peptide control (Figure 6A,B). Western blot analysis suggests that Tat-BECN1 treatment induces autophagy in the chick Chorioallantoic Membrane (CAM) system (Figure 6E and S3H,I).

We proceeded to assess the autophagy inducers SW076956 and SW063058, which have been shown to disrupt the binding of BECN1 to its inhibitory partner, BCL2, without inducing apoptosis [39]. Initially, we examined the effects of dasatinib and SW076956 either as monotherapy or in combination, on autophagy in UMRC-6 cells. Our findings revealed that both agents triggered an increase in autophagy, as evidenced by decreased levels of p62 and lipidated LC3B within the cells, as determined by western blotting (Figure 5A and S4C,D). These observations were further substantiated by a decrease in HiBiT-LC3 luminescence (Figure 5B) and an increase in autophagosome number, as determined using the GFP-LC3 reporter (Figure 5C,D).

Subsequently, we tested the growth of UMRC-6 cells *in vitro* when treated with dasatinib, SW076956, or both together. Remarkably, both compounds significantly inhibited cell growth, with the combination demonstrating a more pronounced effect (Figure S4E). Notably, treatment with rapamycin, an mTORC1 inhibitor, did not affect cell growth in this context (Figure S3E), despite its strong effect on its downstream targets, RPS6K1 and RPS6 (Figure S3D). Next, we performed a high-throughput drug combination screen for dasatinib and SW076956 in UMRC-6 cells. The screen involved testing eight concentrations of each drug in all possible combinations and measuring cell viability three days after treatment. The potential for synergy or antagonism was then analyzed using the Loewe model in Combenefit software [40]. Interestingly, strong synergy was observed between dasatinib and SW076956 with BAP1 inactivation (EV and p.C91S mutant), though to a lesser extent with wild-type BAP1 reconstitution (Figure 5E and S4H). Consistent results were obtained in the high-throughput drug synergy screen of the cholangiocarcinoma cell line TFK-1, with stronger synergism upon BAP1 loss (Figure 5F and S4I).

To generalize our findings to other SRC inhibitors and autophagy inducers, we evaluated the SRC inhibitors bosutinib and saracatinib (Figure S3A). Similar to dasatinib, these drugs dephosphorylated SRC (Figure S3B) and induced autophagy (Figure S3C). Autophagy induction was further increased in combination with the autophagy inducer SW076956 (Figure S4A). Afterward, we performed high-throughput drug combination screens of bosutinib and saracatinib with SW076956 in UMRC-6 and TFK-1 cells. We observed strong synergism in cell viability with saracatinib and SW076956 in TFK-1 cells, but less pronounced synergism with bosutinib and UMRC-6 cells (Figure S5). Furthermore, we observed a synergistic effect when the autophagy inducer SW063058 was combined with dasatinib and saracatinib in TFK-1 cells (Figure S6). However, this synergism was less prominent in BAP1-reconstituted cells.

In summary, these results provide substantial evidence that SRC inhibitors exhibit a synergistic effect in inhibiting cell viability when used in combination with autophagy inducers, particularly in the context of BAP1 loss.

### Autophagy induction in BAP1-deficient cells decreases tumor growth *in ovo*

To evaluate the potential of these compounds as a treatment for BAP1-deficient cancers, we initially implanted UMRC-6 cells subcutaneously in mice. Unfortunately, the cells were unable to grow in mice, which is consistent with a previous report [41]. Therefore, to strengthen the replacement, reduction, and refinement of laboratory animals, we then implanted the UMRC-6 cells into the CAM of fertilized chick eggs. The cells were engrafted in PBS or in PBS containing dasatinib, SW076956, or both. Seven days later, tumors were harvested. Those treated with the combination of dasatinib and SW076956 were significantly smaller than vehicle controls (Figure 6C,D), and western blotting analysis showed induction of autophagy (Figure 6F and S4F,G).

### SRC inhibition and autophagy induction are synergistic in ex vivo preclinical models of patient-derived tumor organoids (PDTOs)

The efficacy of SRC inhibitors and autophagy inducers in treating cancers with loss of BAP1 was further validated through the use of patient-derived tumor organoids (PDTOs) as preclinical models (Table S1). After obtaining written informed consent from patients, we generated PDTOs from fresh ccRCC and UM primary tumors using a modified protocol from a procedure to grow gastrointestinal organoids [42]. Grounded on our previous work with low serum to inhibit mTORC1 [43], we developed a cost-effective conditioned medium by employing low serum conditions. This approach was designed to impede the differentiation and senescence of PDTOs while concurrently preventing the apoptosis in L-WRN cells. We performed a high-throughput drug combination screen with dasatinib and SW076956 in ccRCC and UM PDTOs in at least two independent experiments. The dependence of PDTOs on BAP1 for the synergy of SRC inhibitors with autophagy inducers was measured by the Loewe synergy/antagonism model using Combenefit [40] (Figure S7). Our findings revealed that PDTOs with BAP1 loss exhibited a more pronounced synergy of dasatinib with SW076956 (Figure 6H–J and S7A-C) or saracatinib with SW063058 (Figure S7G,J) compared to PDTOs with wild-type BAP1 (Figure 6K–M and S7). Furthermore, the combination of dasatinib with SW076956 induced autophagy in PDTOs, as indicated by western blotting analysis (Figure 6G and S7M,N). Overall, the PDTOs represent an excellent preclinical platform for testing drug combinations.

## Discussion

Precision oncology has revolutionized cancer therapy by tailoring treatment strategies to an individual’s unique genetic and molecular cancer profile. For cancers with mutations in specific tumor suppressors, these approaches are essential for identifying biomarkers that can aid in early diagnosis and for stratifying patients to receive the most effective treatments. BAP1 is a tumor suppressor that is frequently mutated in deadly cancers, including UM, ccRCC, cholangiocarcinoma and mesothelioma. When mutated, tumors tend to be more aggressive and metastatic. However, treatments for metastatic *BAP1*-mutant cancers are limited. In this study, we describe a novel cell signaling link involving BAP1 loss and the non-receptor tyrosine kinase SRC to block autophagy. This represents a targetable vulnerability in BAP1-deficient tumors.

We describe that BAP1 loss increases the mRNA expression and activation of the proto-oncogene SRC in several cancers, suggesting that BAP1 is a transcriptional repressor of SRC. Indeed, SRC expression is independently associated with poor patient survival in ccRCC and UM. Multiple functions of BAP1 have been described, including transcriptional regulation, double-strand DNA break repair, chromatin modification or maintenance of genomic stability [44], and these different functions are associated with its binding to several transcription factors and regulators. SRC has numerous target proteins involved in a plethora of signaling pathways in the cell regulating tumorigenesis and metastasis, among other functions. Because of the multiple transcription factors or regulators whose activity is known to be regulated by SRC, such as STAT3, STAT5, YAP1, or NF-κB [45], it is plausible to hypothesize a potential feedback loop involving SRC and BAP1, in which nuclear SRC could phosphorylate BAP1, thereby regulating its own transcription.

The BECN1/PI3KC3-C1 complex is a central hub of autophagy regulation and plays an essential role in autophagosome formation [20]. Furthermore, increasingly available data on BECN1 support its central role as a regulator of autophagy via integrating multiple cellular signals through binding with different proteins, post-translational modifications (such as phosphorylation, acetylation, etc), or changes in subcellular localization. Among these signals, the inhibitory effect of growth factors on autophagy was long unclear until the discovery of active EGFR as an interactor and modifier of BECN1 [33]. Active EGFR (both, under stimulating normal conditions and also in active mutants found in cancer) binds to BECN1 and phosphorylates it at tyrosine residues, promoting its homodimerization and inactivation. A similar mechanism of modulating autophagy was described for other tyrosine kinases, such as HER2 [34] and BCR-ABL [35]. Here, we describe a new tyrosine kinase player, the proto-oncogene SRC, as a new (non-receptor) tyrosine kinase regulating autophagy through BECN1 phosphorylation. Although SRC inhibitors were known to induce autophagy [46], the underlying mechanism remained poorly understood. Here, we illustrate that SRC modulates autophagy through BECN1 phosphorylation, which plays an essential role in aberrant cell proliferation in multiple cancer types. We demonstrate that SRC binds to and phosphorylates BECN1, leading to autophagy inhibition dependent on BAP1 status. To our knowledge, this is the first report describing this mechanism of autophagy regulation (besides the regulation of SRC by BAP1). Our data suggest that the inhibitory effect of SRC on autophagy involves the inhibition of the PI3KC3 catalytic subunit, PIK3C3/VPS34. Previous reports have demonstrated that BECN1 phosphorylation at tyrosine residues leads to its homodimerization and decreased binding to VPS34. Thus, it is plausible to hypothesize that a similar mechanism is involved in our experimental settings, as evidenced by increased VPS34 binding to BECN1, as well as increased VPS34 activity in ccRCC cells expressing BAP1, compared to BAP1-deficient cell lines.

The proliferation of BAP1-deficient cells depends on the status of BECN1 tyrosine phosphorylation, which may contribute to tumor progression in certain cancer types. Several reports have indicated that BECN1 regulation of autophagy plays a role in tumor formation. For example, 1) heterozygous deletion of *Becn1* increases the formation of malignancies in mice [47]; 2) BECN1 phosphorylation by AKT1 contributes to fibroblast transformation [48]; 3) increased levels of BECN1 compromise breast xenograft formation *in vivo* [49]; and 4) knock-in mice harboring a BECN1 mutation that prevents binding with its inhibitor BCL2 inhibit HER2+ tumor formation [34]. Additionally, BECN1 contributes to the progression of established tumors by inactivating BECN1 through the overexpression of phosphomimetic mutants in non-small cell lung carcinoma (NSCLC) with activating epidermal growth factor receptor (EGFR) mutations [33], and also HER2+ breast cancers treated with a BECN1-dependent autophagy inducer also inhibit tumor growth [34]. Our data indicate that overexpressing a phosphomimetic mutant of BECN1 in BAP1-deficient UMRC-6 cells significantly inhibits autophagic flux. This overcomes the inducing effects of dasatinib treatment on autophagy and contributes to a significant increase in proliferation *in vitro*. It is difficult to determine whether this effect is due to the role of BECN1 in autophagy regulation or its function in endocytic trafficking. However, our results suggest an essential role for BECN1 in the cellular status in this context.

Treatment with an autophagy-inducing peptide, Tat-BECN1, overcomes the tumor growth and inhibitory effect on autophagy of deregulated receptor tyrosine kinases, such as HER2/ERBB2. Tat-BECN1 promotes autophagic flux in a BECN1-dependent manner. Therefore, treating HER2-positive breast tumorgrafts with the Tat-BECN1 peptide inhibits tumor growth and promotes autophagy [34]. Consequently, we hypothesized that inducing autophagy in BAP1-deficient cells might have a similar effect due to SRC’s inhibitory effect on BECN1. Indeed, our *in ovo* data confirmed this hypothesis in BAP1-deficient ccRCC cells treated with the Tat-BECN1 peptide, as tumor growth was significantly compromised compared to the Tat-scrambled peptide control. These data suggest that autophagy plays a role in tumor growth in this model and could be a target for treatment.

To reduce limitations in the clinical treatment of peptides compared to small molecules, such as stability, tissue penetration, oral bioavailability, manufacturing costs, and immunogenicity risk, as well as to improve specificity compared to other compounds that indirectly promote autophagy, we used the recently developed autophagy inducers SW076956 and SW063058, which are able to disrupt the binding of BECN1 to its inhibitor, BCL2, without inducing apoptosis [39]. Some FDA-approved drugs, like lithium, metformin, and statins, have multiple, often nonspecific, effects that trigger autophagy. However, due to the multiple effects of these drugs, it is difficult to determine if any observed benefits in patients result from the activation of the autophagy machinery or from other mechanisms. Therefore, when identifying compounds that specifically induce autophagy, it is crucial to prove that a compound’s effects are a direct consequence of activating the autophagy machinery. Such a direct and specific effect is illustrated when compounds that specifically induce autophagy are unable to elicit their effects when the autophagy machinery is compromised. Conversely, nonspecific signals that activate autophagy can affect other pathways, including the mammalian target of rapamycin complex 1 (mTORC1), 5’-AMP-activated protein kinase (AMPK), and protein kinase B (AKT). Thus, it is important to distinguish between effects caused by the activation of autophagy and those caused by the modification of other pathways. For instance, BH3 mimetic compounds disrupt the interaction between BECN1 and BECN1 inhibitors of the B-cell lymphoma 2 (Bcl-2) protein family and increase autophagy. However, they also promote apoptosis by interrupting interactions between Bcl-2 family members and BH3 domains of molecules linked to apoptosis. Specific autophagy-inducing agents target regulators of the autophagy process or parts of the molecular autophagy machinery itself, inducing autophagy without affecting other processes, such as apoptosis.

Many clinical trials are testing the combination of autophagy inhibitors with other therapies [24–26]. While autophagy inhibitors may have multiple adverse side effects and toxicities, autophagy inducers may be beneficial for overall cell homeostasis [28]. In fact, mice treated with the autophagy-inducing peptide Tat-BECN1 exhibit no signs of distress during treatment and experience reduced tumor growth [34]. Furthermore, mice with a knock-in mutation in BECN1 that prevents its binding to BCL-2, resulting in constitutively active autophagy, are healthier overall and live longer [50].

Autophagy inducers were tested in our cells alone or in combination with the SRC inhibitor dasatinib, as well as with bosutinib and saracatinib. Although the use of a SRC inhibitor or an autophagy inducer has antiproliferative effects *in vitro* and *in ovo*, a strong synergistic effect was mainly observed in BAP1-deficient cells when combined. Using a more clinically relevant organoid model derived from UM and ccRCC tumors, we observed significant synergy with the combination of dasatinib and SW076956, as well as saracatinib and SW063058, in BAP1-deficient PDTOs but not in wild-type ones. Therefore, tumors with BAP1 loss appear particularly vulnerable to treatments combining SRC inhibitors and autophagy inducers. This strong synergistic effect may be due to the interaction of two control mechanisms that modulate SRC levels. On the one hand is the transcriptional regulation of SRC by BAP1. On the other hand, autophagy also regulates SRC levels, as SRC has been shown to bind to SNX10 and be incorporated into autophagosomes [51]. Consequently, the regulation of SRC protein levels by autophagy may contribute to the observed synergy. While we describe one mechanism involving SRC regulation by BAP1, many others probably regulate the observed phenotype. For instance, we previously found that ccRCC tumors with BAP1 loss exhibit higher mTORC1 activity than wild-type ccRCC tumors or those with *PBRM1* mutations [10]. This can impact various pathways downstream of mTORC1. Additionally, BAP1 has been shown to regulate the expression of the cystine transporter *SLC7A11*, which modulates the levels of reduced glutathione (GSH) and sensitivity to ferroptosis [5]. Altogether, our results suggest a new therapy for tumors with BAP1 loss, regardless of origin, representing a new approach to an unmet clinical need.

**Figure 7.**
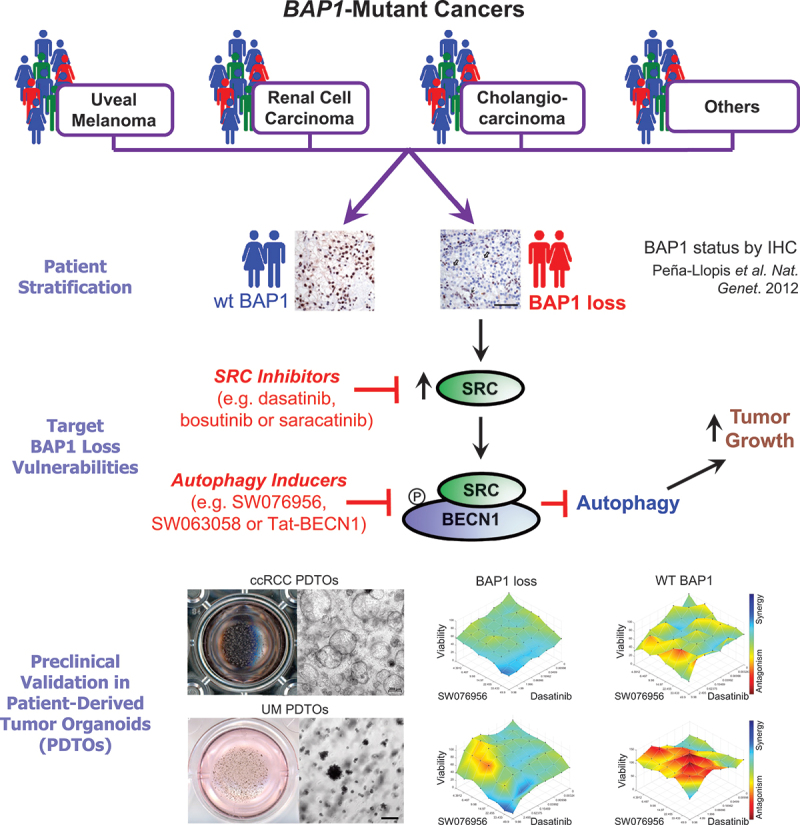
Schematic representation of the proposed mechanism and platform for stratifying BAP1-loss patients who could benefit from treatment with SRC inhibitors and autophagy inducers. Lethal cancers with *BAP1* mutations suppress autophagy through the binding and phosphorylation of BECN1 by the proto-oncogene SRC. Treatments with SRC inhibitors and autophagy inducers exhibited synergism *in vitro*, *in ovo* and *ex vivo* in patient-derived tumor organoids (PDTOs) with BAP1 loss, paving the way for the treatment of BAP1-deficient cancers with a combination of autophagy inducers and kinase inhibitors. The BAP1 immunohistochemistry images were reproduced from [10] with permission from Springer Nature.

Overall, our data indicate the following: 1) BAP1 loss leads to *SRC* upregulation, and BAP1 regulates *SRC* levels transcriptionally; 2) SRC binds to and phosphorylates BECN1, thereby inhibiting autophagy; and 3) treatment of BAP1-deficient tumors with SRC inhibitors and autophagy inducers decreases tumor growth and viability *in vitro*, *in ovo* and *ex vivo* in PDTOs of UM and ccRCC. Furthermore, this drug combination exhibited a synergistic effect. Taken together, our data propose a mechanism whereby tumors with *BAP1* mutations lead to SRC upregulation, allowing SRC to bind and phosphorylate BECN1 and suppress autophagy (Figure 7). Previously, we demonstrated that the BAP1 immunohistochemistry test reliably identifies tumors with *BAP1* mutations in clinical settings [10]. We now provide a rationale for stratifying patients with *BAP1*-mutant cancers for treatment with SRC inhibitors and autophagy inducers. Our data strongly suggest exploring these drug combinations in clinical trials for cancer patients with BAP1 loss, particularly those with metastatic UM, for whom current treatment options are very limited. Additionally, since exercise can induce autophagy [52], consistent endurance training should be recommended to complement pharmacological treatments in patients with BAP1 loss. Our data also open new avenues for treating other cancers with deregulated tyrosine kinases that block autophagy through BECN1 phosphorylation [32], such as EGFR, HER2, BCR-ABL, and c-KIT among others, especially in cases where existing treatments are ineffective or resistance develops to standard therapies. This approach involves using a combination of an autophagy inducer and a specific inhibitor for the particular oncogenic tyrosine kinase.

## Methods

### Cell culture, antibodies and reagents

The ccRCC cell line with a *BAP1* frameshift deletion [10], UMRC-6 (or UM-RC-6), was purchased from Sigma-Aldrich (CB_08090513). The cholangiocarcinoma cell line with a *BAP1* nonsense mutation, TFK-1, was a generous gift from Dr. Stephanie Rössler (University Hospital Heidelberg, Germany). The primary uveal melanoma cell line UPMM2 with a *BAP1* mutation was kindly provided as early passage by Dr. Michael Zeschnigk (University Hospital Essen, Germany) [53]. The primary breast cancer cell line with a nonsense *BAP1* mutation, HCC-1187, was a gift from Prof. Adi Gazdar (UT Southwestern Medical Center, USA). The ccRCC cell line with wild-type *BAP1*, 786–0, was purchased from ATCC (CRL-1932). Human embryonic kidney HEK-293T cells were kindly provided by Prof. Stefan Fröhling (DKFZ, Heidelberg, Germany). All cell lines were maintained in their corresponding medium supplemented with 10% (v/v) fetal bovine serum (FBS) (Gibco 10,500,064) and 1% (v/v) Penicillin-Streptomycin (Gibco 15,140,122) in a humidified incubator at 37°C and 5% CO_2_. The growth medium for UMRC-6 and HEK-293T cells was Dulbecco’s Modified Eagle’s Medium (DMEM) (Gibco 41,965,062); for TFK-1, HCC-1187 and 786–0 cells was RPMI (Gibco 61,870,044); and for UPMM2 was Ham’s F-12 (Gibco 31,765,068). Cells were routinely tested for mycoplasma by PCR. Cell lines were authenticated using Multiplex Cell Authentication by Multiplexion (Heidelberg, Germany) as described [54].

SRC inhibitor Dasatinib was purchased from Biomol and dissolved in DMSO to a stock concentration of 10 mM. Further dilutions in DMSO were made prior to use. Additional SRC inhibitors, bosutinib and saracatinib, were obtained from Biocat and Santa Cruz, respectively, and dissolved at 10 mM in DMSO. Bafilomycin A1 was from MedChemExpress and diluted to 1 mM in DMSO. Tat-Beclin 1 D11 peptide (NBP2–49888) and Tat-Beclin 1 L11S scrambled peptide (NBP2–49887) were purchased from Novus-Biologicals. SW076956 was obtained from D&C Chemicals (Shanghai, China) and dissolved at 50 mM in DMSO. SW063058 was purchased from Merck and dissolved at 2 mM in DMSO. Rapamycin was from ThermoFisher, diluted at 400 μM stock in DMSO. Cycloheximide was from Santa Cruz and diluted in DMSO at 100 mg/ml stock solution.

Antibodies were from the following sources: Santa Cruz (BECN1 for IP [mouse E-8, sc -48,341], Actin-HRP [sc-8432 HRP], total-phospho-Tyr-HRP [sc-7020 HRP]), Millipore (SRC #05–184), Cell Signalling Technology (BECN1 #3738 for WB, phospho-SRC [Tyr416 for mouse, Tyr419 for human] #2101, phospho-SRC [Tyr527] #2105, p62 #88588, HRP-conjugated secondary antibodies [#7074, #7076], phospho-S6K1 [Thr389] #92053, phospho-S6 [S240/244] #22153, total S6K1 #9202, total S6 #2217), Thermo Fisher Scientific (V5 #R960–25), Echelon (VPS34 #Z-R016), BioLegend (HA #901513), Sigma (FLAG M2 #F3165), Bio-Rad (WIPI2 #MCA5780GA).

### Plasmids

V5-tagged (C-terminal) *SRC* (pLX304-*SRC*-V5) was created by Gateway recombination of lentiviral vector pLX304 (Addgene #25890) with pENTR223-*SRC* (CloneID: 170040104) obtained from the Vector and Clone Repository of the Core Facility Cellular Tools at DKFZ, Germany. The plasmids to stably express wild-type *BAP1* (pBABE-hygro-BAP1-HA, Addgene #154020), catalytically-inactive p.C91S mutant *BAP1* (pBABE-hygro-BAP1-C91S-HA, Addgene #154021) or an empty vector control (pBABE-hygro, Addgene #1765) were previously described [29]. Additional BAP1 plasmids were kindly provided by Prof. Boyi Gan [5]: pLVX-M-puro, pLVX-Flag-BAP1, p.C91A, p.S10T, p.E31A, p.L49V, p.H169Q and p.G185R (Addgene #125839–44, 125847, 125849). The pBABE-puro-GFP-LC3 [34], mRFP-GFP-LC3B dual reporter [55] and FLAG-BECN1 (WT, Y229/233/352F, Y229/233/352E) [33] plasmids were previously described. HiBiT-LC3 reporter vector was from Promega [37]. Lentiviral pLKO.1 shRNA *SRC* vectors (TRCN0000038150 and TRCN0000199313) and scrambled control (SHC002) were from Sigma. For *in vitro* kinase assays, the wild-type and kinase-dead (p.K298M) HA-tagged SRC plasmids (Addgene #140294 and #140314) were used.

### Cell line reconstitution with BAP1 constructs

The BAP1-deficient cell lines UMRC-6, TFK-1, and UPMM2 were reconstituted with wild-type *BAP1* (pBABE-hygro-BAP1-HA, Addgene #154020), catalytically-inactive p.C91S mutant *BAP1* (pBABE-hygro-BAP1-C91S-HA, Addgene #154021) or an empty vector control (pBABE-hygro, Addgene #1765), via retroviral infection as previously reported [12,29]. Additional validation was established by transducing the cell lines with wild-type *BAP1* (pLVX-Flag-BAP1, Addgene #125840), several mutant *BAP1* constructs (p.C91A, p.S10T, p.E31A, p.L49V, p.H169Q and p.G185R, Addgene #125841–4, 125847, 125849) or an empty vector control (pLVX-M-puro, Addgene #125839).

### Lentiviral production and transduction of vectors

For stable *SRC* overexpression and/or *SRC* knockdown, lentiviruses were generated in HEK-293T cells cultured in DMEM medium supplemented with 10% FBS and 1% Penicillin-Streptomycin as described [43]. Briefly, HEK-293T cells were transfected with pMD2.G envelope plasmid (Addgene #12259), psPAX2 packaging plasmid (Addgene #12260) and pLKO.1 vectors containing shRNA sequences targeting *SRC*. As control, pLKO.1-Scrambled was used. Virus-containing supernatant was collected and passed through a 0.45 µm syringe filter (Corning 431,220). Cell culture medium of cell lines was replaced with filtered virus-containing supernatant for transduction and cells were selected with 2 µg/ml puromycin (Life Technologies, A1113803).

### siRNA and plasmid transfections

DicersiRNA oligos were obtained from IDT (Integrated DNA Technologies) and transfected with siQUEST (Mirus) according to the manufacturer’s instructions. Assays were performed two days after siRNA transfection. Plasmids were transfected using the Trans-LT1 (Mirus) transfection reagent according to the manufacturer’s instructions, and experiments were performed one day after transfection.

### Generation of HiBiT-LC3 and FLAG-BECN1 cell lines

UMRC-6 cells were seeded into 10 cm^2^ dishes and transfected 24 h later using the corresponding vector: autophagy HiBiT-LC3 reporter vector or FLAG-BECN1 (WT and mutants p.Y229/233/352F, p.Y229/233/352E or empty vector control). Two days after transfection cells were selected with 250 μg/ml of G418 (Gibco) until all cells died in the control, mock-transfected plate. Cells were maintained and expanded in G418, and experiments were performed without G418.

### CRISPR/Cas9 knockout of BAP1 in 786–0 cells

*BAP1* gene was knocked out in the BAP1-competent 786–0 cells using Synthego’s Gene Knockout Kit (Redwood City, CA, USA). Briefly, 786–0 cells were electroporated with sgRNA-Cas9 complexes targeting *BAP1* (sgRNA2 BAP1 + 52408564, U*C*A*AAUGGAUCGAAGAGCGC + Synthego modified EZ scaffold; sgRNA4 BAP1 + 52408601, G*G*A*AGAUAAAUCCAUAUACA + Synthego modified EZ scaffold) or Cas9 control using 4D-Nucleofector X Kit (Lonza, V4XC–2032). Electroporated cells were separated as single cells by limiting dilution and grown into single clones through several rounds of clonal expansion. BAP1 nucleotide deletion was verified by Sanger sequencing and BAP1 loss by western blotting as described below.

### Cell lysates, immunoprecipitation and western blotting

Cells were washed in ice-cold PBS and lysed in ice-cold lysis buffer [56] (Tris-HCl pH7.4, 50 mM, NaCl 250 mM, Igepal 0,5%) containing protease inhibitors (cOmplete Protease Inhibitor Cocktail, Roche or Thermo Fisher Scientific 11,834,101) and phosphatase inhibitors (PhosSTOP Phosphatase Inhibitor Cocktail, Roche or Thermo Fisher Scientific 11,814,101) for 10 minutes at 4°C. Cell debris was depleted by centrifugation at 16,000·*g* for 10 min at 4°C and protein concentration was assessed by Bradford assay. Cleared samples were transferred to a fresh tube, mixed with 4x loading buffer (40% glycerol, 240 mM Tris-HCl pH 6.8, 8% SDS, 0.04% bromophenol blue), containing 10% ß-mercaptoethanol, boiled for 10 min at 95°C and analyzed by Western blotting [29,56]. Samples were loaded into a SDS-PAGE gel and transferred to a 0.45 μm PVDF membrane (Roth). Membranes were blocked 1 h in 5% skim dry-milk in TBST (blocking buffer) and incubated with the corresponding antibodies diluted in 1% BSA-TBST overnight at 4°C. After washing 3 times in TBST membranes were incubated in secondary HRP-conjugated antibodies (1:5000 in blocking buffer) for 45 min at room temperature. Upon additional washing membranes were developed and imaged using FUSION software and imaging system. Western-blot quantification was performed using ImageJ software.

For immunoprecipitation (IP), samples were processed as above but lysed in IP buffer (Tris-HCl pH 7.4 50 mM, NaCl 150 mM, Igepal 0.5% [56]). Lysates were pre-cleared in 30 μl Protein A/G PLUS Agarose beads (Santa Cruz; 1:1 in IP buffer) for 1 h at 4°C. Samples were incubated in the corresponding antibodies (1 μg antibody per mg of protein) overnight at 4°C. 40 μl of Protein A/G PLUS Agarose beads were added for an additional hour and immunoprecipitates were washed three times in IP Buffer, resuspended in loading buffer and boiled for 5 min at 95°C.

### Autophagy assays

Autophagy activity was measured using the following assays: (1) Western blotting analysis of p62 levels and LC3B-I/II ratio [34]. (2) The number of autophagosomes was quantified by measuring the formation of GFP-LC3 puncta. The cells were seeded in a 6-well plate and then subjected to transfection the following day using the pBabe-GFP-LC3 plasmid. Twenty-four hours later, the cells were transferred to a black 96-well plate (Eppendorf) for imaging. Following treatment with 100 nM Bafilomycin A1 (or a DMSO control), cells were fixed in 4% paraformaldehyde (PFA) in phosphate-buffered saline (PBS). The quantification was performed under microscopic analysis, as previously outlined in reference [34]. (3) Quantification of number of autolysosomes/autophagosomes by the RFP-GFP-LC3 reporter was performed similarly as for the GFP-LC3 construct. (4) Luminescence analysis of the HiBiT-LC3 reporter. UMRC-6-HiBiT-LC3 cells were seeded at 4,000 cells/well in triplicate in a white 96 well plate (Santa Cruz). Assays were performed as reported previously [56]. Briefly, 24 h after plating media was changed containing the desired treatment and incubated for the indicated periods of time. Nano-Glo HiBiT Lysis buffer containing LgBiT protein and substrate was added (1:1), incubated in rotation for 10 min at room temperature and luminescence was measured in a plate reader. Parallel plates were set for assessment of the number of cells through Hoechst staining [57] after fixation of the cells in 4% PFA-PBS. (5) Endogenous WIPI2 puncta numbers assessment was performed by immunofluorescence staining with a WIPI2 antibody (Bio-Rad).

### VPS34 activity assay

*In vitro* phosphatidylinositol 3-kinase assay (ADP-Glo kinase assay kit, Promega) was used to determine the activity of VPS34 [58]. Endogenous BECN1 was immunoprecipitated from UMRC-6 cells to isolate the BECN1-bound VPS34 fraction. Immunoprecipitated complexes were washed three times in IP buffer, followed by one wash in TNE buffer (10 mM Tris-HCl pH7.5, 100 mM NaCl, 1 mM EDTA), and resuspended in 60 µl TNE buffer containing 6 µl ATP (0.5 mM in TNE) and 10 µl 100 mM MgCl_2_. Upon addition of the lipid substrate (0.1 mg/ml, V1711, Promega), the mixture was incubated for 20 min at room temperature. Kinase activity was measured using the ADP-Glo kinase assay kit (Promega) according to the manufacturer’s instructions.

### *In vitro* kinase assays

FLAG-BECN1 was purified from UMRC-6 overexpressing cells. HA-SRC was *in vitro* transcribed/translated from a reticulocyte lysate using the TNT T7 coupled Reticulocyte lysate system from Promega, according to manufacturer’s instructions. SRC *in vitro* kinase assays were performed as described [59]. Briefly, purified proteins were mixed in SRC kinase buffer (100 mM Tris-HCl, pH 7.2, 125 mM MgCl_2_, 25 mM MnCl_2_, 2 mM EGTA, 250 µM Na_3_VO_4_, 2 mM DTT) containing 100 µM ATP and incubated at 30°C for 30 min. Samples were analyzed by western blotting.

### Cell proliferation

Cells were plated in 96-well plates and eight hours later a plate was fixed in 4% PFA-PBS as a time 0 sample. Cells were treated with the required treatments and fixed in 24 h intervals. At the end of the sampling period, plates were washed with PBS, fixed for 10 min at room temperature, and stained with Hoechst diluted in PBS for 20 min at room temperature in the dark. Fluorescence intensity was measured in a BioTek plate reader.

### Colony formation assay

UMRC-6 cells (2,500 per well) were seeded in triplicate in 2 mL of culture medium in 6-well cell culture plates. Uniform distribution of individual cells was ensured by microscopy. Assay plates were incubated at 37°C and 5% CO_2_ for 3 weeks. The culture medium was refreshed every 3–5 days. After 3 weeks, the culture medium was aspirated and the cells were washed with cold PBS (Thermo Fisher Scientific 14,190,169) and placed on ice. For fixation, 1 ml of cold methanol (J.T. Baker, Phillipsburg, NJ, USA, 8045) was added per well and incubated for 10 min on ice with gentle agitation. The methanol was removed and 1 ml of 0.1% crystal violet (Sigma-Aldrich, St. Louis, MO, USA, C0775) in 25% (v/v) methanol was added and incubated for 30 min at room temperature with gentle agitation. The staining solution was removed, the wells were washed thoroughly with tap water and the plates were air dried upside down on tissue paper. Plates were scanned using an Epson Perfection V850 Pro scanner (Epson, Suwa, Japan) and the associated Epson Scan software. Confluency was quantified using the ImageJ ColonyArea plug-in as previously described [60].

### SRC protein stability

UMRC-6 cells were incubated with 50 µg/ml cycloheximide at different time points and harvested for western blotting analysis.

### Semi-automatic drug combinations in cell lines

Compounds were dissolved in DMSO to create a concentration of 10 mM and were dispensed into white-bottom sterile polystyrene 384-well plates (Corning, 3570) at nine concentrations ranging from 5 nM to 50 µM in triplicate, using a Tecan D300e Digital Dispenser (Tecan, Switzerland/HP Inc., CA, USA) and D300e Control software [12]. Negative controls with only DMSO were also included. To ensure capturing the linear growth phase of cells during drug treatment, we optimized cell number and assay period by seeding each cell line in triplicate at ten different cell densities, ranging from 100 to 3,000 cells per well, in 30 µl of cell culture medium in nine replicate assay plates, and performing a cell viability assay every 24 h for eight days. Cell viability was analyzed by normalizing luminescence values to day 0. For drug combination experiments, 500 cells per well were seeded in 30 µl of culture medium using a Multidrop Combi dispenser (Thermo Fisher Scientific, MA, USA) and assay plates were incubated at 37°C and 5% CO_2_ for 4 days (96 h for bosutinib and saracatinib) or 5 days (120 h for dasatinib). The CellTiter-Glo assay (Promega) was performed as previously described [12] to determine cell viability using a Tecan Spark 10M, a Berthold TriStar 2 (Berthold Technologies, Bad Wildbad, Germany) or a Promega GloMax Explorer microplate reader.

### Generation and culture of patient-derived tumor organoids (PDTOs)

Surgical resections of ccRCC and UM primary tumors were collected at the University Hospital Essen, Germany, after ethical approval by the Faculty of Medicine of the University of Duisburg-Essen (19–8553-BO and 21–10013-BO) and written informed consent was obtained from all patients. Patient-derived tumor organoids (PDTOs) were generated from fresh tumors as previously described [42] with some modifications. Briefly, primary tumors of ccRCC and UM were minced into very small pieces (1–2 mm^3^) and dissociated in Dissociation Solution containing 1 mg/ml dispase (Life Technologies 17,105,041) and 1 mg/ml collagenase Ia (Sigma-Aldrich, C9891) in RPMI for 15 min on ice and 50 μg/ml DNase I (Roche 11,284,932,001) for 15 min at 37°C. Organoids were cultured and maintained in Growth Factor Reduced Matrigel (Corning 356,253) or Cultrex Reduced Growth Factor Basement Membrane Extract (Bio-Techne, 3536–005–02). A conditioned medium from L cells that express Wnt3a, R-spondin-3 and Noggin (L-WRN) [61,62] was used with some modifications. We collected the medium of L-WRN cells grown in 0.1% FBS (but also possible up to 1% or even 5% FBS instead of 10% FBS of the original conditioned medium) to prevent the differentiation and senescence of PDTOs and at the same time to prevent the apoptosis of L-WRN cells when grown in a serum-free medium, based on our previous studies in low serum conditions [43]. The organoid medium is enriched with the supplements enumerated in Table S2. The resulting final concentration of FBS in the organoid medium is less than 0.025%, low enough to allow almost unlimited passaging of PDTOs, while providing enough serum to prevent apoptosis of L-WRN cells and allow the release of Wnt3a, R-spondin-3 and Noggin.

Organoids in culture were monitored microscopically and passaged periodically to maintain optimal growth conditions. For organoids generated from fresh tumor samples, expansion may occur after 7–10 days of incubation, while intermediate and late passages may require up to 21 days of incubation. The success rate is also influenced by the inherent characteristics of the organoids, with patterns varying between different organoids. To passage PDTOs, the organoid medium in the 24-well plates was discarded. Each well was washed with 500 µl of 1x PBS (Life Technologies 14,190,094) and then treated with 500 µl of Cell Recovery Solution (Corning 354,253). The plate was incubated on ice with a rocking platform in a 4°C cold room for 1 hour to liquefy the extracellular matrix gel. The mixture of extracellular matrix gel and organoids was then pipetted into a 15-ml tube (Sarstedt, 62.554.502), and Advanced DMEM F-12 medium (Life Technologies 12,634,028) supplemented with 1% penicillin-streptomycin (10,000 U/ml) (Life Technologies 15,140,122), 1% 1 M HEPES (Life Technologies 15,630,056), and 1% GlutaMAX (Life Technologies 35,050,038) was added to a final volume of 10 ml.

For full 24-well plates of organoids, 12 wells were pipetted into each 15 ml tube to ensure thorough washing. The washing steps were repeated two more times until the organoid pellet was completely separated from the matrix gel. The organoid pellet was then mixed with fresh liquid matrix gel, seeded into new 24-well plates, and incubated for 1 h in a 37°C incubator before adding organoid medium. All of these steps were performed on ice except for the initial step of creating the dome organoids.

### Drug combinations in PDTOs

Drug treatments in ccRCC and UM PDTOs were conducted in 384-well plates (Corning, 4588) as described elsewhere [63] using 10% Basement Membrane Extract (BME). PDTOs were treated with seven concentrations of dasatinib or saracatinib, seven concentrations of SW076956 or SW063058, and their combinations in triplicate for 3 days (ccRCC PDTOs) or 4 days (UM PDTOs) and organoid viability was assessed by CellTiter-Glo 3D as described [12,63] using a Tecan Spark 10M, a Berthold TriStar 2 (Berthold Technologies, Bad Wildbad, Germany) or a Promega GloMax Explorer microplate reader. Two to three independent experiments were analyzed for synergistic effects.

### Western blotting for PDTOs

PDTOs in 24-well plates were washed in ice-cold PBS and incubated in 500 µl of Cell Recovery Solution for 30 min on ice. Resuspended PDTOs were centrifuged at 3,000·*g* for 5 min at 4°C and washed once with PBS. The pelleted PDTOs were then lysed in lysis buffer on ice and proteins were prepared for western blotting as described above (see Protein Lysate section).

### TCGA analysis

Reverse phase protein array (RPPA) and RNA-Seq data from ccRCC and UM patients from The Cancer Genome Atlas (TCGA) were downloaded from the Genomic Data Commons (GDC) data portal (https://portal.gdc.cancer.gov). Updated mutation and survival data for KIRC-TCGA and UVM-TCGA were obtained [14,64] and analyzed as previously described [29,65].

### ChIP-Seq analysis

Raw ChIP-Seq data were obtained from Gene Expression Omnibus (GEO) repository with accession numbers GSE40723 [30] and GSE65555 [66] and analyzed with Integrative Genomics Viewer (IGV) (Broad Institute, https://software.broadinstitute.org/software/igv) using mm9 genome.

### Quantitative reverse-transcribed PCR (qRT-PCR)

RNA extraction and qRT-PCR analysis were performed as described previously [12]. *SRC* mRNA expression was quantified using forward 5’-TGGCAAGATCACCAGACGG-3’ and reverse 5’-GGCACCTTTCGTGGTCTCAC-3’ primer pairs, p62/*SQSTM1* mRNA using forward 5’-GCACCCCAATGTGATCTGC-3’ and reverse 5’-CGCTACACAAGTCGTAGTCTGG-3’ primer pairs and normalized to the housekeeping genes peptidylprolyl isomerase B (*PPIB*) using forward 5’-GAGGAAAGAGCATCTACGGTG-3’ and reverse 5’-GCTTCTCCACCTCGATCTTG-3’ primer pairs [12] or *B2M* using forward 5’-TGCTGTCTCCATGTTTGATGTATCT-3’ and reverse 5’-TCTCTGCTCCCCACCTCTAAGT-3’ primer pairs.

### Chromatin immunoprecipitation and qPCR (ChIP – qPCR)

ChIP was performed using the ChIP-IT Express Enzymatic Kit (Active Motif) according to the manufacturer’s instructions. Briefly, UMRC-6 cells stably transduced with FLAG-tagged BAP1 or empty vector were cross-linked with a final concentration of 1% formaldehyde (Sigma) for 10 min. Collected cells were lysed and the nuclear pellets were resuspended in a shearing cocktail, incubated for digestion at 37°C for 10 min. Shearing efficiency was assessed by agarose gel electrophoresis, and DNA concentration was quantified to normalize the amount of chromatin for ChIP. FLAG antibody or IgG (Sigma) was used to immunoprecipitate the chromatin samples. qPCR for *SRC* promoter region occupancy was quantified in a qTower^3^ G (Analytik Jena, Germany) as described above using forward 5’-GACCCCCAGCAAGCCAG-3’ and reverse 5’-GGAGTTGAAGCCTCCGAACA-3’ primer pairs.

### *In ovo* chick chorioallantoic membrane (CAM) assay

Freshly fertilized eggs were incubated at 38°C with 50% atmospheric humidity in a humidified rotary incubator. On embryonic day 10, eggs were candled shining light into the eggshell to identify the chorioallantoic vein and a square was marked 1 cm away from the branching point. A little hole was made on the blunt end of the egg causing the air to escape allowing the “upper” chorioallantoic membrane to sag creating an artificial air chamber. This was followed by the opening of a 1 × 1 cm window on the eggshell where the square was drawn. Immediately after opening the window, “the upper CAM” was lightly scuffed using a cotton tip. Then 50 μl of cell suspension (1.5 × 10^6^ cells in PBS) were graphted onto the CAM membrane. The window was then sealed with tape to avoid contamination or drying of the CAM and the eggs were kept at the incubator for another 7 days to allow tumor development. On day 17 post-fertilization eggs were sacrificed and the chicken was killed by decapitation after 15 min of ice incubation. Eggs were cut longitudinally and washed with PBS at least twice. Tumor identification was made following morphological (unified mass formation differentiated from other membrane components) and angiogenesis features (increased vasculature formed surrounding the tumor). Tumors were extracted, measured and cut in two for both histological (4% PFA in PBS fixation) and western blotting analysis (flash frozen in liquid nitrogen) as described above.

For protein extraction of CAM tumors, flash frozen pieces were homogenized in three volumes of lysis buffer containing protease and phosphatase inhibitors on ice as described [67]. Homogenized samples were lysed for 30 min at 4°C and further cleared with a QIAshredder column (Qiagen) by centrifugation at 16,000·*g* for 10 min at 4°C. Extracted proteins were analyzed by western blotting following the standard protocol.

### Statistical analysis

Groups were compared and *p* values calculated using a two-tailed unpaired Student’s *t* test when equal variances or a Welch’s *t* test when unequal variances. One-way analysis of variance (ANOVA) with Student-Newman-Keuls (SNK) *post-hoc* tests were calculated using IBM SPSS Statistics 29.0, where non-overlapping letters (e.g., “a” vs. “b”) represent significant differences (*p* < 0.05). Microsoft Excel and GraphPad Prism 9 and 10 were used to calculate the other statistical analyses.

## Supplementary Material

KAUP_2024_0311R3_Supplementary_Material.docx

## Data Availability

The data that support the findings of this study are included in this published article and its supplementary information files. Materials on this study are available from the corresponding authors on reasonable request and might require a material transfer agreement.
