# Supplementary material for: Autophagy suppression via SRC induction represents a therapeutic vulnerability for BAP1-mutant cancers
Source: Autophagy. 2025 Aug 3;21(12):3324–43. doi: 10.1080/15548627.2025.2535265 (PMC12758184; doi:10.1080/15548627.2025.2535265)
Supplement: KAUP_2024_0311R3_Supplementary_Material.docx [file KAUP_A_2535265_SM4387.docx]

Supplementary Material for

**Autophagy suppression via SRC induction represents a therapeutic vulnerability for *BAP1*-mutant cancers**

Silvia Vega-Rubin-de-Celis^1,*^, Amanda Kristani^2,3,4^, Matthias Kudla^1^, Svenja Mergener^2,4^, Andrés Corrochano-Ruiz^2,4^, Safa Larafa^1^, Jetsy Montero-Vergara^1^, Laura-Marie Ahle^3,4^, Rainer Will^5^, Mael Lever^3^, Viktor Grünwald^6,7^, Boris Hadaschik^6^, Verena Jendrossek^1^, Nikolaos E. Bechrakis^3^, Samuel Peña-Llopis^2,3,4,*^

^1^Institute for Cell Biology (Cancer Research), University Hospital Essen, Hufelandstrasse 55, D-45147 Essen, Germany.

^2^Translational Genomics, German Cancer Consortium (DKTK) at University Hospital Essen, Essen, Germany.

^3^Department of Ophthalmology, University Hospital Essen, Essen, Germany.

^4^German Cancer Consortium (DKTK) and German Cancer Research Center (DKFZ), Heidelberg, Germany.

^5^Core Facility Cellular Tools, German Cancer Research Center (DKFZ), Heidelberg, Germany.

^6^Department of Urology, University of Duisburg-Essen, and German Cancer Consortium (DKTK), University Hospital Essen, Essen, Germany.

^7^Department of Medical Oncology University Hospital Essen, Essen, Germany.

*These authors contributed equally to this work.

Corresponding authors: [Silvia.VegaRubindeCelis@uk-essen.de](mailto:Silvia.VegaRubindeCelis@uk-essen.de)

[Samuel.Pena-Llopis@uk-essen.de](mailto:Samuel.Pena-Llopis@uk-essen.de)

**Table S1. Clinico-pathological features of Patient-Derived Tumor Organoids (PDTOs).**

| **Patient ID** | **Gender** | **Age at Surgery (y)** | **Primary Tumor** | **BAP1 status** | **Pathology Primary Tumor (T)** | **Pathology Lymph Node (N)** |
| --- | --- | --- | --- | --- | --- | --- |
| UKE-RCC-03 | Female | 65 | ccRCC | Loss | pT2b | pNX |
| UKE-RCC-04 | Male | 44 | ccRCC | WT | pT3a | pN0 (0/4) |
| UKE-RCC-09 | Male | 46 | ccRCC | WT | pT1a | pN0 (0/8) |
| UKE-UM-02 | Male | 71 | UM | Loss | pT4b | pNX |
| UKE-UM-05 | Female | 65 | UM | WT | pT3b | pNX |
| UKE-UM-10 | Male | 54 | UM | Loss | pT3b | pNX |
| UKE-UM-11 | Female | 72 | UM | WT | pT2a | pNX |
| UKE-UM-13 | Male | 69 | UM | WT | pT3b | pNX |
| ccRCC, clear-cell renal cell carcinoma; UM, uveal melanoma. Pathology Primary Tumor (T) and Lymph Node (N) are part of the TNM staging system to describe the size, extent and invasiveness of the primary tumor. | | | | | | |

| **Component** | **Final Concentration** | **Supplier** | **Catalog Number** |
| --- | --- | --- | --- |
| Human FGF-basic (FGF-2/bFGF) (154 aa) Recombinant Protein | 0.01 µg/ml | Peprotech | 100-18B-250 |
| Human FGF-10, Animal-Free Recombinant Protein | 0.01 µg/ml | Peprotech | AF-100-26-100 |
| Human EGF, Animal-Free Recombinant Protein | 0.05 µg/ml | Peprotech | AF-100-15-100 |
| Prostaglandin E2 | 1 µM | Peprotech | 3632464 |
| A83-01 | 0.5 µM | Peprotech | 9094360 |
| SB202190 | 0.5 µM | Peprotech | 1523072 |
| Nicotinamide | 4 mM | Santa Cruz | sc-208096A |
| Bovine Serum Albumin | 0.1 mg/ml | Sigma-Aldrich | A7906 |
| B-27 Supplement (50x) | 1x | Gibco | 17504044 |
| N-2 Supplement (100x) | 1x | Gibco | 17502048 |
| Penicillin-Streptomycin (10,000 U/ml) | 0.2x | Life Technologies | 15140122 |
| GlutaMAX | 0.2x | Life Technologies | 35050038 |
| HEPES | 2 mM | Life Technologies | 15630056 |

**Table S2. Composition of Organoid Medium for Patient-Derived Tumor Organoids (PDTOs).**

**Figure S1. SRC is upregulated in BAP1-deficient tumors and cell lines.**

**Figure S1. SRC is upregulated in BAP1-deficient tumors and cell lines.** (**A**) RPPA data analysis of clear-cell renal cell carcinoma (ccRCC) patients from TCGA (KIRC-TCGA) with *BAP1* mutations compared to patients with *PBRM1* mutations. (**B,C**) Quantification of western blot data of SRC protein levels in ccRCC UMRC-6 cells (**B**) and uveal melanoma (UM) UPMM2 cells (**C**). Representative western blot analysis of SRC levels in the cholangiocarcinoma cell line TFK-1 (**D**) and breast cancer cell line HCC-1187 (**F**), and quantification analysis of multiple experiments (**E,G**). (**H,I**) *SRC* gene expression (**H**) and SRC protein expression (**I**) are negatively correlated with BAP1 expression in RPPA from KIRC-TCGA. (**J,K**) SRC protein stability was determined upon cyclohexamide treatment (50 µg/ml) at the indicated times in UMRC-6 cells reconstituted with BAP1 or an empty vector control (EV). Data shown are a representative western blot (**J**) and quantification analysis of several experiments (**K**). (**L**) Kaplan-Meier analysis in UM patients with intermediate (Int) or high levels of *SRC* expression exhibited poorer overall survival than patients with low levels of *SRC* in UVM-TCGA. (**M**) ChIP-Seq occupancy of FLAG-BAP1 and the multiprotein complex partners HCF-1 and OGT on the *Src* promoter. Data represent the average ± SE of three independent experiments. *, *P*<0.05; **, *P*<0.01; *t*-test. Non-overlapping letters (e.g., "a" *vs*. "b") represent significant differences (*P*<0.05) using ANOVA with Student-Newman-Keuls test.

**Figure S2. Autophagy is inhibited in BAP1-deficient cells.**

**Figure S2. Autophagy is inhibited in BAP1-deficient cells.** Autophagic activity in TFK-1 cholangiocarcinoma cells was analyzed by western blotting (**A**), HiBiT-LC3 luminescence (**B**) and number of GFP-LC3 puncta (*n* = 50) (**C**). Autophagic activity in uveal melanoma cells UPMM2 reconstituted with WT-BAP1 or a p.C91S mutant (or an empty vector control, EV) was analyzed by western blotting (**D**) and number of GFP-LC3 puncta (*n* = 50) (**E**). Autophagic activity of HCC-1187 cells was determined by western blotting (**F**) and HiBiT-LC3 luminescence (**G**). (**H**) BAP1-competent ccRCC 786-0 cells depleted of BAP1 using two different CRISPR-Cas9 sgRNAs were analyzed by western blotting. Where indicated, starvation with HBSS was for 3 h, and bafilomycin A1 (BafA1) was at 100 nM for 3 h. (**I**) qRT-PCR of *SQTSM1* mRNA expression in the indicated UMRC-6 cells. Western blot (**J**) and qRT-PCR (**K**) of UMRC-6 cells treated with Bafilomycin A1 (BafA1; 100 nM) for the indicated times. Quantification of p62 (**L**) and LC3B-II (**M**) protein levels in UMRC-6 cells expressing the indicated *BAP1* mutants treated with Bafilomycin A1 (100 nM, 3h) or DMSO (vehicle). Data represent the average ± SE of three independent experiments. *, *P*<0.05; **, *P*<0.01; ***, *P*<0.001; *t*-test. Non-overlapping letters represent significant differences (*P*<0.05) by ANOVA with Student-Newman-Keuls test.

**Figure S3. SRC inhibitors induce autophagy in BAP1-deficient cells.** (**A**) Chemical structure of SRC inhibitors (left) and autophagy inducers (right) from PubChem. (**B**) Western blot of UMRC-6 cells treated with the indicated compounds for 24 h (dasatinib, 500 nM; torin, 100 nM). (**C**) Autophagic activity in UMRC-6 cells treated with 500 nM dasatinib, 10 µM saracatinib, 10 µM bosutinib or DMSO control for 24 h in combination with 100 nM Bafilomycin A1 (or DMSO control) for 3 h was analyzed by the number of GFP-LC3 puncta (*n* = 50). (**D**) Representative western blot of mTORC1 activity markers on UMRC-6 cells treated with 100 nM rapamycin for the indicated times. (**E**) Cell proliferation of UMRC-6 cells treated with two concentrations of rapamycin for the indicated time. (**F,G**) Autophagic activity of UMRC-6 cells treated with Tat-BECN1 peptide (TB) or Tat-Scrambled control (TS) (10 µM, 3 h) was assessed by HiBiT-LC3 luminescence (**F**) and western blotting (**G**). (**H,I**) Quantification of western blot analysis of UMRC-6 cells xenografts from fertilized chick eggs in a chorioallantoic membrane (CAM) assay and treated with 10 µM Tat-BECN1 peptide (TB) or Tat-Scrambled control (TS) for 7 days. (**J,K**) Colony formation of UMRC-6 cells overexpressing the indicated BECN1 constructs or an empty vector (EV) control (**J**) and its quantification (**K**). Data represent the average ± SE. *, *P*<0.05; **, *P*<0.01; *t*-test. Non-overlapping letters represent significant differences (*P*<0.05) by ANOVA and Student-Newman-Keuls test.

**Figure S4. Synergistic effect of dasatinib and SW076956 in BAP1-deficient cells.**

**Figure S4. Synergistic effect of dasatinib and SW076956 in BAP1-deficient cells.** (**A**) HiBiT-LC3 luminescence of UMRC-6 cells treated with the indicated compounds for 24 h (500 nM dasatinib, 10 µM saracatinib, 10 µM bosutinib and/or 40 µM SW076956). (**B**) Treatment effects of dasatinib and Tat-BECN1 on cell proliferation in the BAP1-deficient cell line UMRC-6. UMRC-6 cells were plated in a 96-well plate at 1000 cells/well. The next day, cells were treated with 30 µM of Tat-Scrambled or Tat-BECN1 peptide diluted in acidified OPTI-MEM media for 2 h. Full DMEM media containing 12.5 or 25 nM dasatinib (or DMSO control) was then added to the plate. Plates were fixed at the indicated times with 4% PFA-PBS for 10 min, washed with PBS and then stained with Hoechst for 20 min. The number of nuclei per well were automatically assessed in a Cytation5 microscope. (**C,D**) Quantification of p62 (**C**) and LC3B-II (**D**) protein levels of UMRC-6 cells treated with the indicated compounds for 24h with or without Bafilomycin A1 (100 nM, 3h). (**E**) Cell proliferation of UMRC-6 cells treated with dasatinib (500 nM), SW076956 (40 µM) or a combination of both (D+SW) for the indicated times. (**F,G**) Quantification of western blot analysis of p62 (**F**) and LC3B-II (**G**) from UMRC-6 xenografts derived from fertilized chick eggs in a chorioallantoic membrane (CAM) assay treated with dasatinib and/or SW076956 for 7 days. (**H,I**) Synergy/antagonism effects of dasatinib and SW076956 were determined by a Loewe synergy model using Combenefit software from three independent experiments in UMRC-6 (**H**) and TFK-1 cells (**I**). Data represent the average ± SE. *, *P*<0.05; **, *P*<0.01; ***, *P*<0.001; *t*-test. Non-overlapping letters indicate significant differences (*P*<0.05) by ANOVA with Student-Newman-Keuls test.

**Figure S5. Synergistic effects of bosutinib and saracatinib with SW076956 in BAP1-deficient cells.** Combination effects of bosutinib and saracatinib with SW076956 treatments on cell viability of UMRC-6 (**A**,**E**) and TFK-1 (**B**,**F**) cells were quantified 72 h after treatment of serial dilutions of single and combined compounds in three (**A**,**E**) or four (**B**,**F**) independent experiments. Synergy/antagonism effects of bosutinib and saracatinib with SW076956 were determined by a Loewe synergy model using Combenefit software of three independent experiments in UMRC-6 (**C**,**G**) and four independent experiments in TFK-1 cells (**D**,**H**). *, *P*<0.05; **, *P*<0.01; ***, *P*<0.001.

**Figure S6. Synergistic effects of dasatinib and saracatinib with SW063058 in TFK-1 cells.** (**A**,**C**) Combination effects of dasatinib (**A**) and saracatinib (**C**) with SW063058 treatments on cell viability of TFK-1 cells reconstituted with an empty vector (EV), wild-type *BAP1* or p.C91S *BAP1* mutant were quantified 72 h after treatment of serial dilutions of single and combined compounds in at least three independent experiments. (**B**,**D**) Synergy/antagonism effects of dasatinib (**B**) and saracatinib (**D**) with SW063058 were determined by a Loewe synergy model using Combenefit software from at least three independent experiments in TFK-1 cells. *, *P*<0.05; **, *P*<0.01; ***, *P*<0.001.

**Figure S7. Synergistic effects of dasatinib and SW076956 (as well as saracatinib and SW063058) in BAP1-deficient PDTOs from ccRCC and UM.** (**A**-**F**) Synergy/antagonism effects of dasatinib and SW076956 were determined by a Loewe synergy model using Combenefit software in a ccRCC PDTO with BAP1 loss (three independent experiments) (**A**), two UM PDTOs with BAP1 loss (two independent experiments) (**B**,**C**), a ccRCC PDTOs with wild-type BAP1 (two independent experiments) (**D**), two UM PDTOs with wild-type BAP1 (two independent experiments) (**E**,**F**). (**G**-**L**) Combination effects of treatment with different concentrations of saracatinib and SW063058 on the viability of a ccRCC PDTOs with BAP1 loss (two independent experiments) (**G**,**J**), a UM PDTO with wild-type BAP1 (four independent experiments) (**I**,**L**), and a ccRCC PDTO with wild-type BAP1 treated with dasatinib and SW076956 (two independent experiments) (**H**,**K**). (**M,N**) Quantification of western blot analysis of p62 (**M**) and LC3B-II (**N**) from a representative ccRCC PDTO to validate the induction of autophagy by the combination of dasatinib and SW076956 in PDTOs.
